# Learning time-varying information flow from single-cell epithelial to mesenchymal transition data

**DOI:** 10.1371/journal.pone.0203389

**Published:** 2018-10-29

**Authors:** Smita Krishnaswamy, Nevena Zivanovic, Roshan Sharma, Dana Pe’er, Bernd Bodenmiller

**Affiliations:** 1 Department of Genetics, Department of Computer Science, Yale University, New Haven, CT, United States of America; 2 Institute for Molecular Life Sciences, University of Zurich, Zurich, Switzerland; 3 Department of Applied Physics and Applied Math, Columbia University, New York, NY, United States of America; 4 Program for Computational and Systems Biology, Sloan Kettering Institute, Memorial Sloan Kettering Cancer Center, New York, NY, United States of America; University of South Alabama Mitchell Cancer Institute, UNITED STATES

## Abstract

Cellular regulatory networks are not static, but continuously reconfigure in response to stimuli via alterations in protein abundance and confirmation. However, typical computational approaches treat them as static interaction networks derived from a single time point. Here, we provide methods for learning the dynamic modulation of relationships between proteins from static single-cell data. We demonstrate our approach using TGFß induced epithelial-to-mesenchymal transition (EMT) in murine breast cancer cell line, profiled with mass cytometry. We take advantage of the asynchronous rate of transition to EMT in the data and derive a pseudotime EMT trajectory. We propose methods for visualizing and quantifying time-varying edge behavior over the trajectory, and a metric of edge dynamism to predict the effect of drug perturbations on EMT.

## Introduction

Cellular identity is largely determined by the computations occurring in a cell: what inputs does a cell sense, how it processes these inputs through regulatory networks and how it implements a response. Responses to environmental cues play a key role in development, cellular differentiation and fate. Different cellular states therefore have altered input-output behavior. A useful analogy is to imagine a cell as a logic circuit, with a clearly defined input-output mapping. In a cell, gene and protein interactions form the logic. If the input to the circuit changes then the intermediate signals (levels of genes and proteins) may change, but the underlying circuitry is the same. What truly defines a cellular identity change, such as during differentiation, development, or cancer progression is the reconfiguration of the logic itself.

In this work, we test and utilize this intuitive understanding by quantifying the rewiring of the regulatory network along a progression of cells in single-cell data. We computationally align the cells into a one-dimensional trajectory, also known as “pseudotime” in literature [1–3] to approximate the progression of cells along a real time axis, and study protein interactions along it. We demonstrate our approach using the epithelial-to-mesenchymal transition (EMT), which is a controlled state change system that naturally occurs during embryogenesis and cancer progression but can be induced artificially. EMT can be initiated by an external TGFß signal, resulting in signaling and transcriptional activation, followed by functional and morphological changes. We study EMT using a mouse breast-cancer cell line [4, 5] measured with mass-cytometry. TGFß-induced EMT is thought to involve the SMAD, MAPK and AKT pathways, which activate multiple transcription factors such as Snail1, Slug, Twist and Zeb and in turn their targets [6, 7], thereby altering the underlying regulatory network and response to input stimuli. An example of altered input-output behavior in EMT is in cell adhesion signaling. Epithelial cells can sense cell-cell adhesion and grow in response to that, while mesenchymal cells do not process this information. This can be seen in the decrease in E-Cadherin expression level, indicative of loss of cell-cell contact, as E-Cadherin is an archetypal protein that mediates cell-cell adhesion [8].

In order to assess the rewiring of network, we quantify the change in association strength between pairs of proteins (edges) during the EMT process. Such edges are based on statistical dependencies and reflect direct or indirect phosphorylation or other specific biological mechanisms. In particular, we first approximate the EMT progression by aligning cells onto a one-dimensional trajectory using wanderlust [1], followed by quantification of changes in edge strength continuously along the pseudotime. The construction of the pseudotime is facilitated by the asynchronous nature of the transition and the availability of cells in all phases of EMT on a single snapshot of data. To track signal strength along the wanderlust derived trajectory, we extend our previously developed mutual-information based metric for quantifying edge strength DREMI (conditional-Density Resampled Estimate of Mutual Information) [9] into a metric to model the functional dependence of a protein on another protein and pseudotime. In particular, for a given pair of assayed proteins X and Y, we model each cell with three coordinates (1) pseudotime of the cell (2) abundance of X (3) abundance of Y, and by treating each cell as a point of information we learn modulation in relationship between X and Y along pseudotime. We call our metric Trajectory Interpolated DREMI Scores (TIDES) and use it to analyze the ebb and flow of information in the network. We find that edges involved in EMT change in their strength throughout the transition. We validate our edge modulation assessment using perturbations that support our TIDES metric.

We therefore have a new dynamic network model where each edge has (pseudo)-time varying strength. This description of the network is significantly different than previous dynamic models of edges such as Ordinary Differential Equations (ODEs) [10, 11] and has different advantages. ODEs describe the model as being governed by differential equations where the dynamics themselves are fixed, i.e., they carry a quasi steady-state assumption. Thus, they cannot be directly applied to find the ways in which the network is rewired or actively reprogrammed over time. Other approaches such as Bayesian networks [12] provide a static picture of the network and are not able to quantify how a network changes over time. However, the advantage of the time-varying view of a network is that most state changes such as EMT are likely defined by a cascade of network reconfigurations before reaching a final state. That is, different gene modules are turned off and on as differentiation occurs and this cannot be encoded as a time invariant system based on measured variables.

If this is indeed the case, then a promising avenue for preventing undesirable end states is to disrupt this rewiring process itself by inhibiting edges that are critical to the process as defined by some measure of criticality, a notion we address in this manuscript. For this, we also develop an edge criticality measure that takes into account the overall dynamism of the edge. We validate this hypothesis with another type of perturbation: a chronic inhibition of the protein kinase for 5 days and observe the fraction of cells transitioned at the end of 5 days as an endpoint measurement, thus proving that our time varying view of the regulatory network is amenable for finding perturbations, and potentially therapeutic targets.

## Results

### Measuring signaling during TGFß-induced EMT

**Fig 1:**
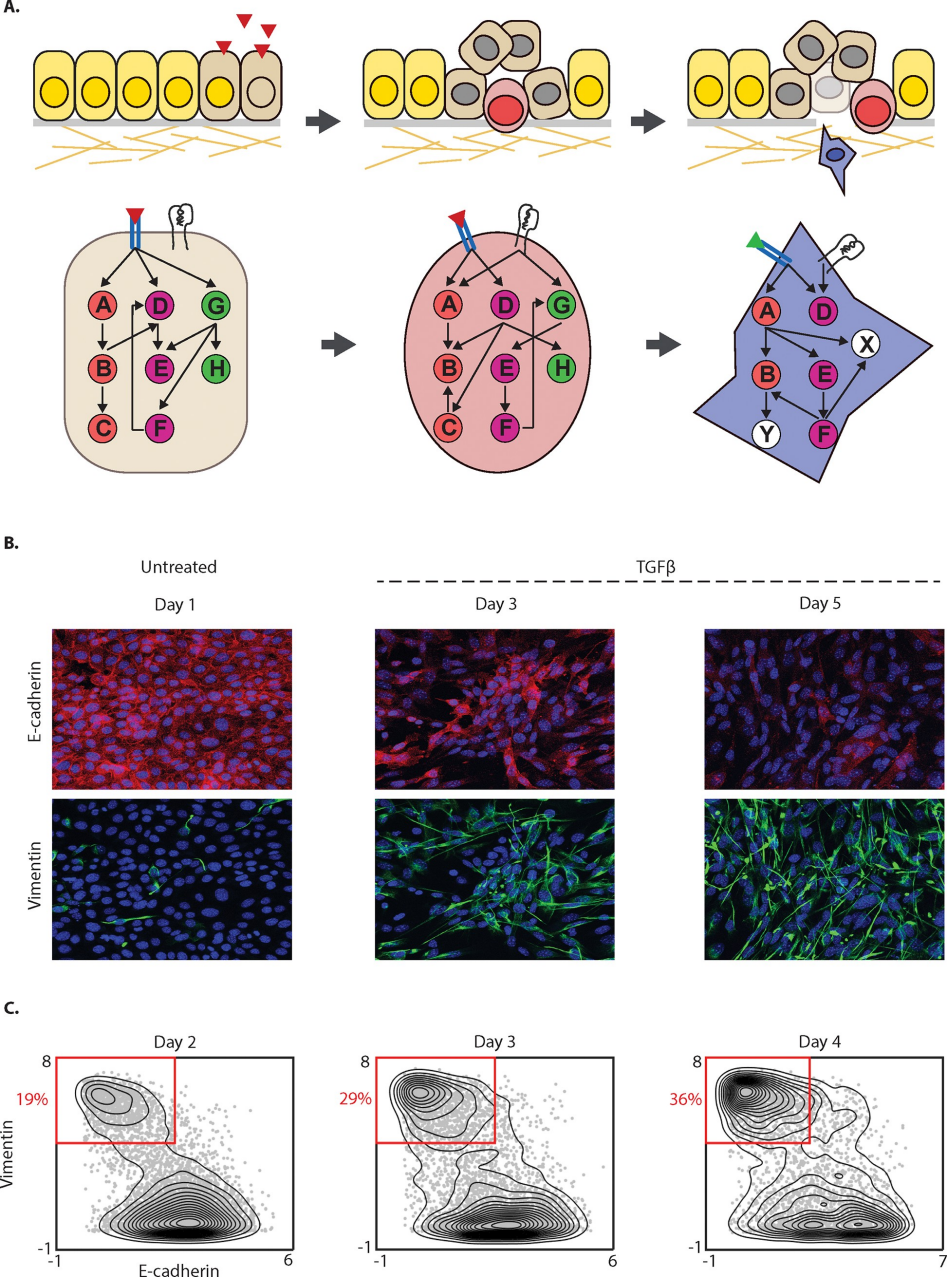
**Edge modulation during phenotypic change in EMT.** (A) Conceptual diagram of edge modulation as cells undergo EMT. (B) Immunofluorescence images of Py2T cells stained for canonical markers E-cadherin (in red) and Vimentin (in green) are shown after 1, 3 and 5 days of 4ng/ml TGFß stimulation. Three days after TGFß treatment we find both cells that express E-cadherin and cells that express Vimentin. (C) Contour plots of Vimentin and E-cadherin following 2–4 days of TGFß exposure show a shift in density from epithelial to mesenchymal with 19%, 29% and 36% of cells in the mesenchymal phase respectively. The data is arcsinh transformed with a cofactor of 5. The plots show a continuum of intermediate cell states indicating that EMT is a rate-heterogeneous process.

To study the signaling network and phenotypic changes during EMT (Fig 1A), we used Py2T murine breast cancer cells following chronic exposure to TGFß [13] (Fig 1B). Cells were sampled daily in biological triplicate over a four-day TGFß time course. We used mass cytometry [14] to assay transcription factors and signaling activity (phospho-protein abundance) in single cells. A total of 32 markers were simultaneously measured, including three surface markers and 29 intracellular markers (S1 Table). The markers were chosen to assess epithelial (high expression of E-cadherin) and mesenchymal (high expression of Vimentin and CD44) states, signaling activity of the SMAD, AKT, MAPK, WNT and NFκB pathways, EMT transcription factors, cell cycle, and apoptosis (S1 Table).

**Fig 2:**
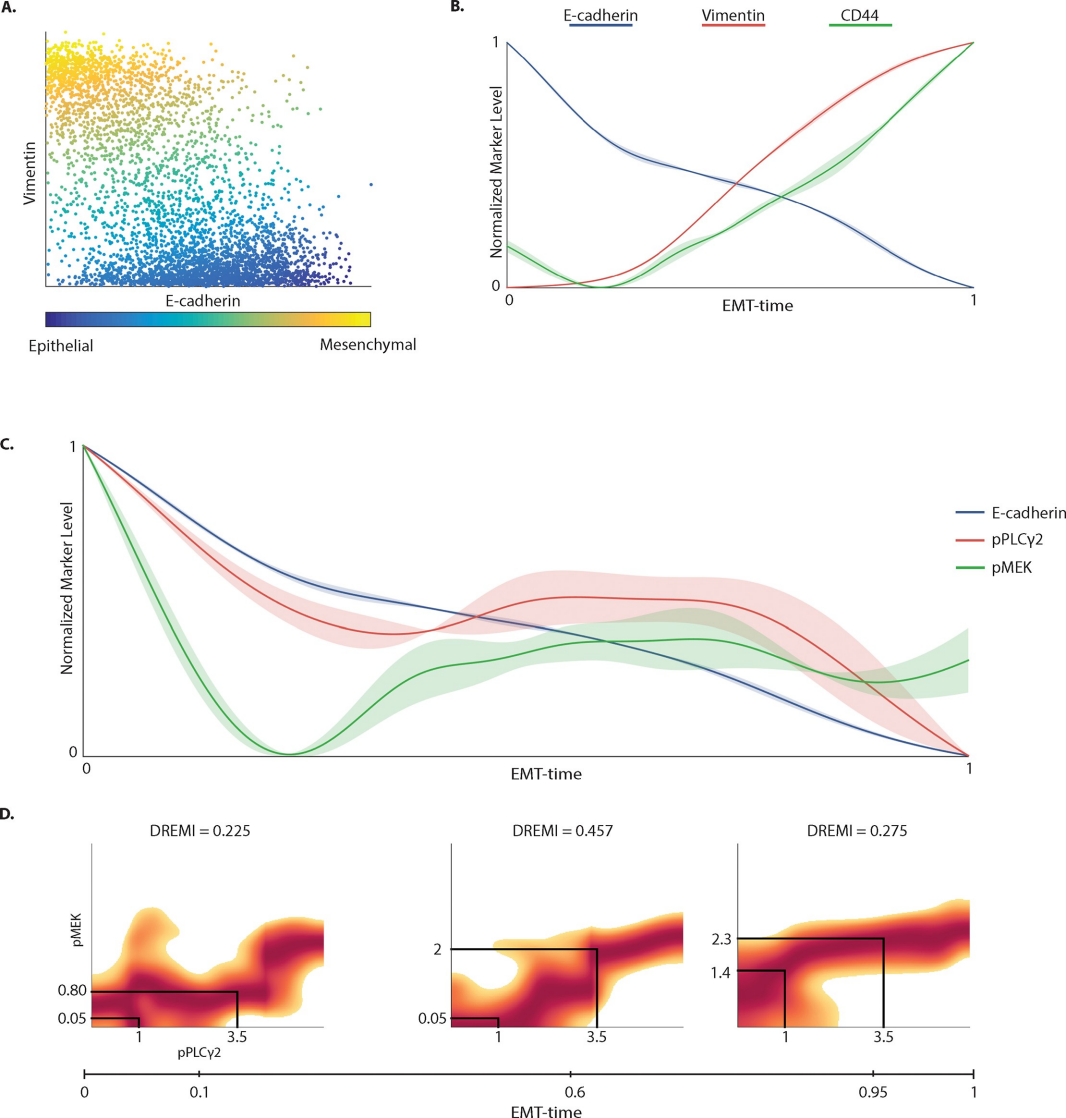
**Expression of molecules along EMT-time (wanderlust pseudotime).** (A) Scatterplot where each point represents a Py2T cell collected 3 days after TGFß stimulation, colored by their Wanderlust-derived pseudotime label, which we call “EMT-Time” [1]. (B) Smoothed expression levels of E-cadherin, Vimentin, CD24 and CD44 along EMT-time. The EMT-time is normalized to a scale of 0–1, where epithelial cells are near 0 and mesenchymal cells are near 1. Marker levels are also normalized to 0–1 and are smoothed using a sliding-window Gaussian filter. The shaded region around each curve captures 1-standard deviation across replicates, indicating consistency. (C) Smoothed expression levels of signaling markers pPLCγ2 and pΜΕΚ1/2, as well as E-cadherin along EMT-time. (D) DREVI (conditional-Density Rescaled Visualization) [9] plots show the relationship between pPLCγ2 and pMEK1/2 at three different points along EMT-time corresponding to epithelial, transitional and mesenchymal phenotypes. Each DREVI plot illustrates the renormalized conditional density estimate of the abundance of pMEK1/2 given the abundance of pPLCγ2. The red color indicates the conditionally dense regions. The solid black lines indicate that an equal rise in the level of pPLCγ2 results in a higher increase in the abundance of pMEK1/2 during the transitional phase as compared to the epithelial and mesenchymal phase. The strength of the relationship is quantified by DREMI, which computes mutual information on the conditional probability between two molecules.

Starting on day two, we observed a profound number of cells ranging from the epithelial to the full mesenchymal state. Days two, three and four had 19%, 29% and 36% of cells in the mesenchymal state (Fig 1C, Parts A and B of S1 Fig). The epithelial cells showed high levels of E-cadherin. Cells labeled as mesenchymal recapitulated mesenchymal characteristics, including loss of E-cadherin and gain of Vimentin. The transitioning cells exhibited intermediate marker expression that shared both epithelial and mesenchymal characteristics, based on the expression of E-cadherin, CD44 and Vimentin (Parts A-E of S2 Fig). Taken together, we observed a continuum of cells from the epithelial to the mesenchymal state that implies a high-degree of variability either in the rate of transition or in commitment of a given cell to the EMT state. Therefore, rather than treating EMT as a two state-system, in all subsequent analyses we treated the heterogeneous population of cells as a continual trajectory and ordered cells along a pseudotime axis of EMT progression, inferred using the Wanderlust algorithm [1]. We call the Wanderlust pseudotime ordering “EMT-time” (Fig 2A and 2B).

### Extracting an EMT progression from static mass cytometry data via wanderlust

Given multi-dimensional single cell data, Wanderlust infers a one-dimensional axis of progression and has been shown to recapitulate developmental trajectories [1, 15]. We applied Wanderlust separately to cells from days two, three and four after EMT induction based on E-cadherin and Vimentin. EMT-time recapitulated expected changes: E-cadherin showed a monotonic decrease in abundance while Vimentin and CD44 showed a monotonic increase through the transition (Fig 2B and Parts A-C of S3 Fig). The marker expression trend is robust across replicates (mean cross-correlation > 0.86) (Part D of S3 Fig). Moreover, the inferred trajectories are similar at different days following EMT induction. Part E of S3 Fig shows that the Wanderlust trajectories are closely correlated between days 2, 3 and 4 (mean cross-correlation > 0.76), suggesting that EMT-time might represent a cell-state that is agnostic to the day of measurement, once the full range of cells from the epithelial to the mesenchymal state are present.

### Signaling edges along EMT progression

Using the EMT progression, we studied the modulations of pairwise relationships in the data or edges in the signaling network. Such relationships are based on statistical dependencies. Studying the dynamics of protein expression and protein phosphorylation levels can tell us which pathways are modulated. Studying an *edge* over time can tell us about how influences between molecules and pathways change. We first compared a canonical edge pPLCγ2-pMEK1/2 between the epithelial, transitional and mesenchymal states using DREMI and DREVI [9] to quantify and visualize edge strength. Fig 2C and 2D illustrates how the expression of pMEK1/2 and pPLCγ2 and the relationship between them changes along EMT-time. The DREMI score between pPLCγ2 and pMEK1/2 increases from 0.225 to 0.457 from the epithelial to transitional state, and subsequently decreases to 0.275 as the cells approach the mesenchymal state (Fig 2D). Thus, the abundance of pPLCγ2 holds most information on pMEK1/2 levels in the intermediate state: the same increase in the abundance of pPLCγ2 (from 1 to 3.5, in the units of arc-sinh transformed protein abundance) corresponds to only a small increase in the abundance of pMEK1/2 in epithelial cells (from 0.05 to 0.8) and mesenchymal cells (from 1.4 to 2.3), but a higher increase in transitional cells (from 0.05 to 2).

**Fig 3:**
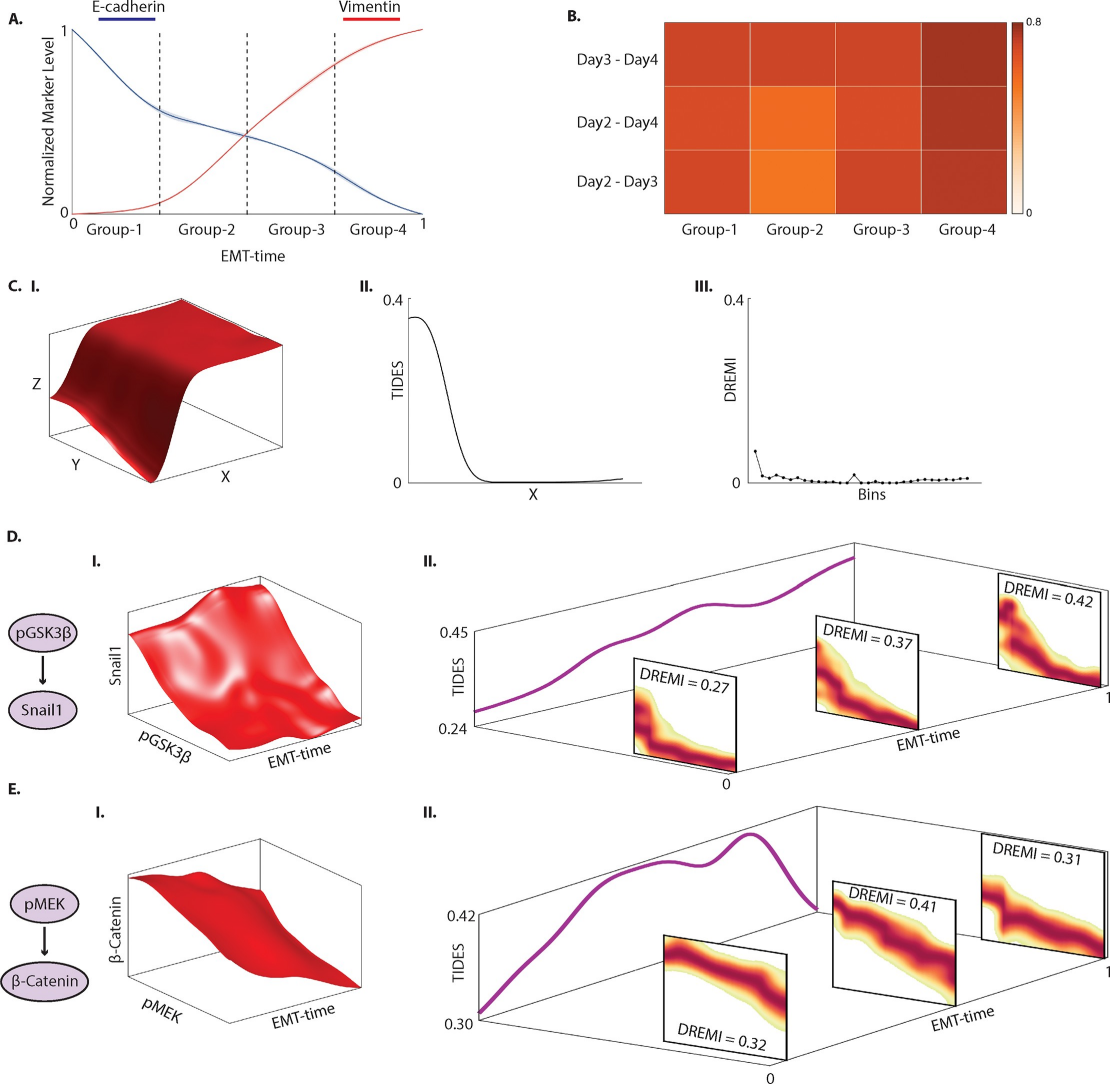
**Signaling relationships along EMT-time.** (A)-(B) Relationship between signaling molecules is similar across days when controlled for EMT-time. (A) TGFß-treated cells from Days 2, 3 and 4 are binned into four groups along EMT-time. Expression levels of E-cadherin and Vimentin are shown for reference. (B) Heat map shows the correlation of DREMI scores computed on all pairs of signaling molecules in each group across days. The mean correlation is 0.69. (C) A simulated example illustrating transient relationships captured by TIDES. (I) 3D-DREVI representation of a simulated data with three variables T, X and Y. X and Y have strong but transient relationship at lower values of T, which flattens out as T increases. (II) TIDES of X-Y at various values of T. Continuous nature of TIDES allows it to correctly detect the strong but short-lived relationship between X and Y at lower values of T. (III) DREMI scores of X-Y for overlapping bins along T. Binning the data is unable to capture any relationship between X and Y. (D)(I) 3D-DREVI between pGSK3ß and Snail1 along EMT-time on Day 3. (II) The pseudo-dynamics of the relationship between pGSK3ß and Snail1 along EMT progression is represented by the TIDES curve (purple curve) which shows the time-varying change in relationship strength (depicted on the Z axis) in the units of DREMI. The 2D-DREVI slices depict the normalized conditional density estimate of the abundance of Snail1 given the abundance of pGSK3ß at three specific time-points during EMT. (E) (I) A 3D-DREVI plot of the relationship between pMEK1/2 and ß-Catenin on Day 3. (II) The TIDES curve and slices of 2D-DREVI along EMT progression show the dynamics of the pMEK1/2—ß-Catenin relationship.

We sought to confirm whether signaling relationships were more dependent on the actual time point after TGFß induction of EMT (wall time), or on a cell’s position in the EMT progression as derived by Wanderlust (EMT-time). The latter is a possibility when different cells progress at individual rates through a fixed EMT program. We binned cells into four stages based on our inferred EMT-time (Fig 3A). For each bin, we computed DREMI scores for all pairs of signaling proteins. We found a high mean correlation of 0.69 between the DREMI scores across days (Fig 3B) when controlled for phase-of-transition (i.e., bins along EMT-time). This result also holds true across various replicates (Part A of S4 Fig). This result suggests that in our experimental system many signaling relationships are determined by the phase, whereas differences in behavior between time points (wall time) in bulk measurements largely derive from the different proportions of cells in each phase.

Since our data indicates a continuous trajectory with transitional cells between the epithelial and mesenchymal states, we formulated a method to model how relationships between proteins continuously rewire over the course of the EMT progression. We selected Day 3 as a representative sample where cells were relatively uniformly spread throughout the transition. This sets the stage for analysis of protein signaling relationships and their dynamics during the EMT cell-state transition from a single snap-shot.

### Inferring information modulation in edges

Given a particular signaling edge X-Y, the relationship involves X processing the information it receives (cues via upstream proteins) and passing it onto Y via biochemical modifications such as phosphorylation. This can be thought of as informational flow from X to Y. As a cell is undergoing a drastic cell state transition such as EMT, such relationships could have different behavior or strengths depending on where the cell is during the transition. Therefore, it is of interest to gain a continuous view of informational flow in an edge. To this end, we extended DREVI to a 3^rd^ dimension, where the level of the molecule Y is modeled as a function of two parameters: the abundance of the molecule X and EMT-time (T) (See Methods). DREVI is based on the empirical conditional density, estimated directly from the data. As dimensionality increases, data becomes sparser and therefore robust density estimation becomes more challenging. We extended the heat-equation based kernel density estimation [16] used in [9] to higher dimensions (See Methods). We then normalize the density estimate by *two* parent dimensions, rather than one dimension as in [9], to derive the conditional distribution on an X-T plane. We typically visualize a red surface representing the conditionally dense portion of DREVI surface that shows Y’s “typical” behavior for each level of X and point T along EMT-time (Fig 3CI). Once the ***3D-DREVI*** is computed, we can compute ***3D-DREMI***, measuring the degree of information X *and* T together provide for the value of Y, analogous to 2D-DREMI [9] (see Methods).

While 3D-DREMI provides a general score indicating the degree in which both X and T influence Y, it does not directly address how edge strength changes over time. To derive a quantification of the change in edge strength over the course of the trajectory we introduce a new dynamic measure of dependency that we call *T**rajectory* *I**nterpolated* *D**R**E**MI* *S**cores (TIDES)*. A TIDES curve is computed by first calculating a 3D conditional density estimate *f*(Y|X, T) where T is EMT-time and X-Y are two molecules whose time varying dependency we intend to assess. Next, we linearly interpolate the 3D conditional density at a fixed value of T (EMT-time) to obtain a 2D slice of the relationship between X and Y (See Fig 3CI, 3D and 3E, Methods for details). Thus, the projection of the 3D-conditional density on to a slice allows us to compute the DREMI score between the two markers at any given EMT-time. When taking a causal interpretation of an edge (possibly due to prior knowledge of mechanism), higher DREMI suggests that X exerts a stronger influence on Y. Computing DREMI at each point along EMT-time results in a TIDES curve, which provides a concise, quantitative view describing how pairwise molecular relationships change during the progression.

The continuous nature of TIDES enables the detection of transient relationships that could potentially be critical but hard to detect computationally. For example, Fig 3CI shows a simulated example of three entities T, X and Y where X and Y have a transient but strong relationship for small values of T. The relationship then weakens as the value of T increases (indicated by the flattening of 3D-DREVI surface). This short-lasting relationship is correctly captured by TIDES (Fig 3CII) because it allows for density to be continuously interpolated along T. However, an alternative approach in which we computed DREMI in discrete overlapping bins (Fig 3CIII) was unable to detect the transient interaction.

### A continuous view of edge modulation during EMT

TIDES allows us to examine how the relationship between two molecules evolves during a state transition. For example, the relationship between signaling molecule GSK3ß and the transcription factor Snail1 is shown in Fig 3DI. GSK3ß phosphorylates Snail1 at two motifs and is known to inactivate its transcriptional activity and cause protein degradation [6]. However, phosphorylation of GSK3ß (pGSK3ß) (e.g. through the AKT and PKC pathways [17]) inhibits its activity and therefore pGSK3ß is positively correlated with Snail1. Snail1, in turn, modulates genes relevant to EMT and among others activates additional transcription factors [6]. The strength of the relationship between pGSK3ß and Snail1 is weak at the beginning of the transition (DREMI = 0.27) and then grows steadily and peaks as the cells are on the verge of completing the transition (DREMI = 0.42), Fig 3DII. This change is consistent across replicates (Part B of S4 Fig).

Another example is the edge between phosphorylated MEK (pMEK1/2) and ß-Catenin shown in Fig 3E. This relationship increases and peaks in strength during the transition (DREMI = 0.41) and decreases as the transition concludes (DREMI = 0.31). It is known that MEK1/2-ERK1/2 pathway is initiated by activation of Ras mediated by ShcA in response to TGFß treatment [6]. Activated MEK1/2-ERK1/2 pathway can then directly phosphorylate LRP6, which is a co-receptor and a key regulator of the WNT/ß-Catenin signaling pathway [18, 19]. We find that this interaction is transmitting the most information during the transitional phase, as indicated by the high DREMI score. This change is consistent across replicates (Part C of S4 Fig).

**Fig 4:**
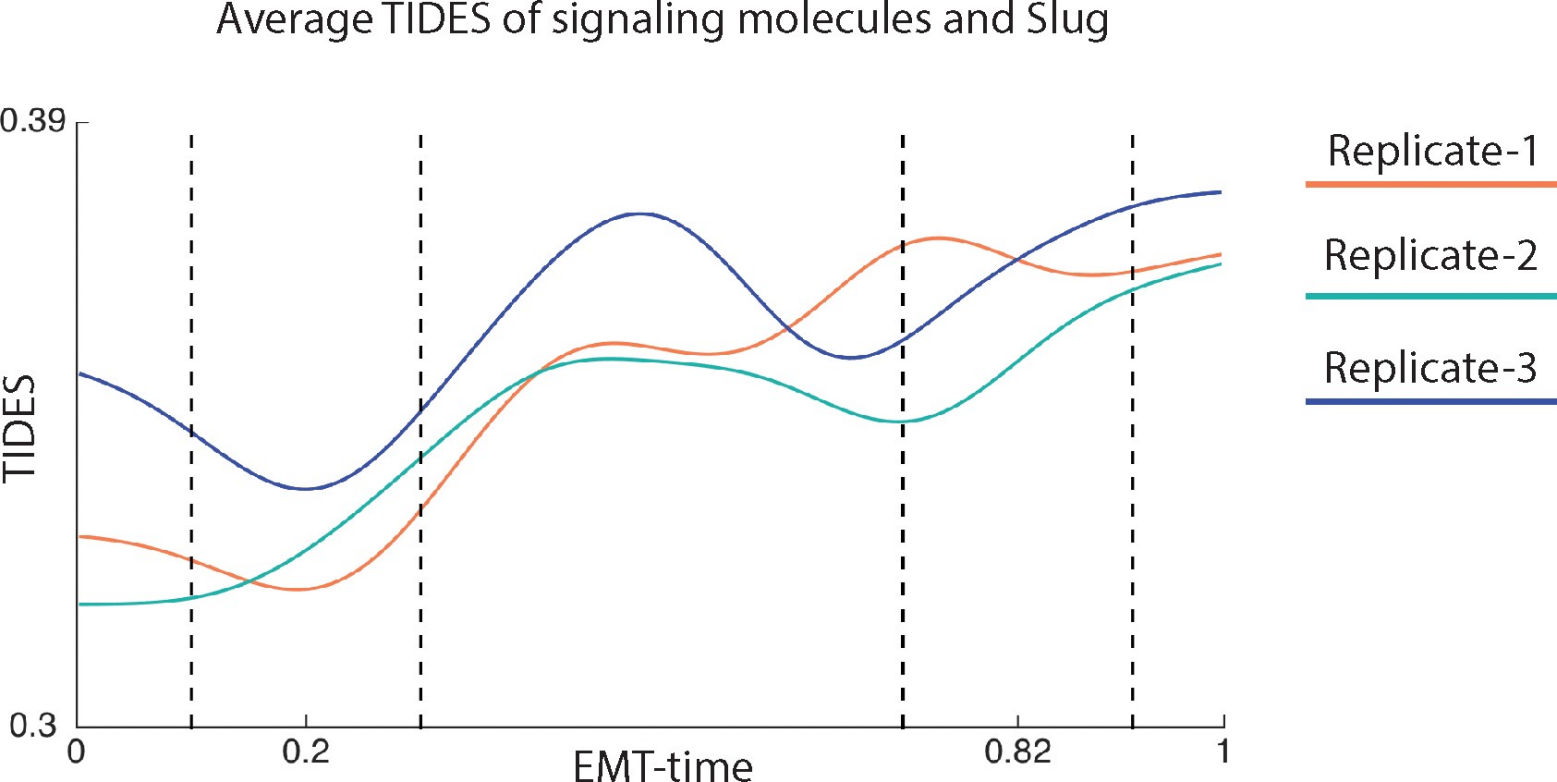
**Information transfer during EMT.** Average TIDES curve of the relationship between several molecules (pCREB, pSTAT5, pFAK, pMEK1/2, pNFκB, pP38, pAMPK, pAKT, pERK1/2, pGSK3ß, pSMAD1/5, pSMAD2/3, ß-catenin, CAHIX, pMARCK, pPLCγ2, pS6, pSTAT3) and Slug, across three replicates for Day 3. The curves start rising steadily at near EMT-time = 0.20, and peak near EMT-time = 0.82.

In addition to analyzing edges individually, TIDES can also be used to globally understand when there is a high information flow in the entire system. There are points in EMT-time when many signaling molecules pass signal to transcription factors (points of high DREMI). It can be assumed that such points of high information transfer correspond to critical points, when the system is going through a phase transition. In Fig 4, we combine TIDES scores incoming into the EMT transcription factor Slug, a known core-regulator of EMT [20] which in turn regulates additional EMT transcription factors such as Twist [21]. The average TIDES curve of signaling molecules into Slug shows the scores start rising at around EMT-time 0.20, around when the cell morphology begins to change and hence corresponds to where the transition is beginning. We see a peak towards the end of the transition at around EMT-time 0.82 which might correspond to an additional change in cell state (Fig 4). Additionally, we see similar behavior at similar EMT-times for two more EMT transcription factors, Snail1 and Twist (Parts A and B of S5 Fig).

### Validation of TIDES with acute inhibitions

**Fig 5:**
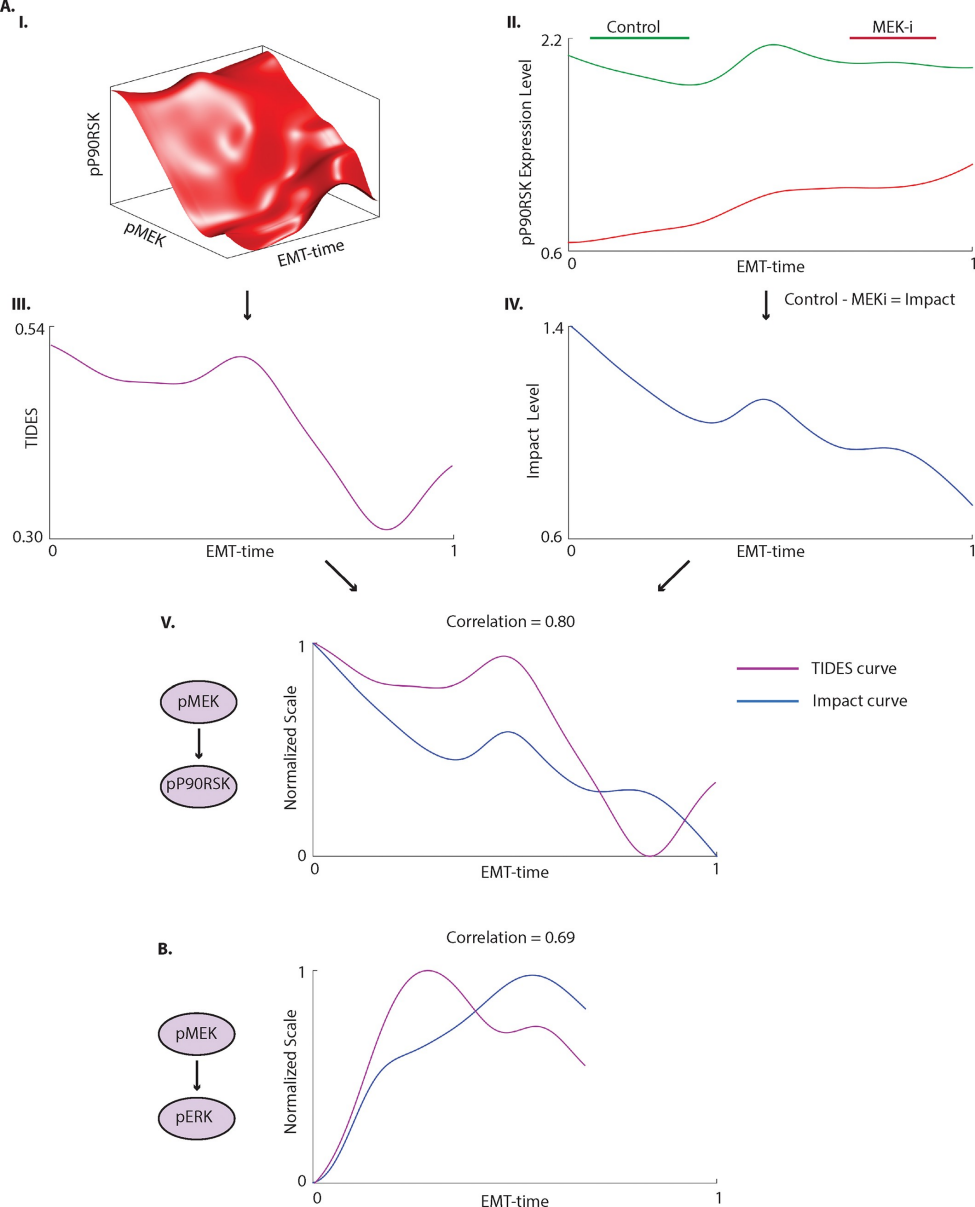
**Validation of TIDES via short-term drug inhibition.** (A) (I) 3D-DREVI plot shows a typical behavior of pP90RSK given pMEK1/2 and EMT-time. The cells are treated with TGFß for 3 days. (II) The levels of pP90RSK under control (stimulated with TGFß) and under MEK1/2-inhibition (TGFß + MEK1/2i) along EMT-time. As expected, MEK1/2-inhibition substantially reduces the level of pP90RSK as compared to the control. (III) TIDES curve between pMEK1/2 and pP90RSK. (IV) The impact curve, computed as the level of pP90RSK under control minus under MEK1/2-inhibition, shows regions of high effect of MEK1/2-inhibition on pP90RSK along EMT-time. (V) Cross-correlating the curves results in a correlation of 0.80. The depicted curves have been normalized to 0–1 and shifted appropriately based on the lag obtained from cross-correlation (see Methods). (B) Cross-correlating the TIDES curve of pMEK1/2 on pERK1/2 against the impact curve of pERK1/2 under MEK-inhibition gives a correlation of 0.69.

TIDES quantifies the strength of relationship between proteins at any point during the transition. For a given pair of proteins *X-Y*, high TIDES value suggests a stronger influence of *X* on *Y* and a lower TIDES value indicates a weaker influence. This motivates the idea that upon inhibition of *X*, assuming *X*-*Y* is the causal direction, the expression level of *Y* should be impacted more in regions of high TIDES as compared low TIDES. We define an *impact curve* as the difference between the abundance of Y along EMT-time under control (no drug-perturbation) and the abundance of Y along EMT-time with drug-perturbation of *X*. We expect regions of high TIDES to coincide with the regions of high impact and test this by correlating the TIDES curve against the impact curve, using cross-correlation to match the trajectories (Fig 5A and Methods).

For the ease of interpretation, we consider relationships between proximal members along a short signaling pathway. Distant relationships can have inputs or convergence from several pathways and therefore the TIDES curve may not accurately match the output. To this end, we chose to inhibit MEK1/2 because it has a potent and specific inhibitor and we were able to measure proximal downstream phosphorylation targets of MEK1/2 (ERK1/2 and P90RSK) by mass cytometry. Inhibiting the kinase for 30 minutes should accentuate the immediate downstream effects on signaling pathways without substantially altering transcriptional activity, EMT phenotype, or allowing for compensatory effects. Hence, we can directly compare EMT-time of the control and treated condition.

Py2T cells were treated with TGFß for 3 days, followed by inhibition of MEK1/2 by the small molecule PD184352 for 30 minutes. We first compared the pP90RSK impact curve along with the pMEK1/2-pP90RSK TIDES curve, Fig 5A. pMEK1/2 is upstream of pP90RSK with pERK1/2 the mediatory kinase that directly phosphorylates P90RSK. We find that the impact curve shows a high cross-correlation of 0.80 with the TIDES curve (Fig 5A(V)), a trend that is repeated across replicates (Part A of S6 Fig). Note the correlation between the abundance of pP90RSK in control and the TIDES curve is only -0.01 demonstrating that 1) TIDES does not trivially follow levels of the Y-molecule, and 2) that it adds additional predictive value to edge strength (Part B of S6 Fig). Similarly, the impact curve of pERK1/2 under MEK1/2-inhibition matches the pMEK1/2-pERK1/2 TIDES curve with a cross-correlation of 0.69 (Fig 5B, Part C of S6 Fig), further validating the approach. The cross-correlation between the pERK1/2-pP90RSK TIDES curve and the impact curve of pP90RSK under MEK1/2-inhibition is also high (0.74 and 0.59 across replicates, Parts D and E of S6 Fig). Thus, we have validated the predictive capability of TIDES in measured edges downstream of pMEK1/2 in our data. This validation suggests that TIDES successfully predicts the impact to downstream partners in signaling relationships and can therefore be used to study the time-varying behavior of signaling edges.

### Identification and validation of driver edges in EMT via 3D-DREMI

Next, we wanted to identify edges that are potential drivers for EMT based on edge modulation behavior. We hypothesized that driving edges should involve proteins that have a strong dependence on *both* EMT-time, and each other for the following reasons: a). A protein that is not influenced by where a cell is during a transition is less likely to play important role during the transition b). Proteins that do not interact strongly with each other are less likely to work together to drive the transition. In other words, we speculated that proteins involved in edges with high 3D-DREMI along the pseudotime play more important role in driving EMT. We wanted to make sure that for a given pair of molecules X-Y and EMT-time (T), X and T *together* provide more information about Y as compared to individually and Y is also highly dependent on both X and T, individually. Therefore, we add together a 3D-DREMI score on (T, X)-Y *and* 2D-DREMI on X-Y and T-Y in our panel and sort them by their average score across the three replicates. We find that the top ranking edges are pSMAD2/3 –ß-catenin, ß-catenin–pSMAD23, pGSK3ß –pERK1/2, pAMPK–pSMAD23, pAMPK–ß-catenin, pGSK3ß –ß-catenin, pERK–ß-catenin and pMEK1/2 –pAMPK (S3 Table). This suggests that these proteins, and their corresponding pathways, could be involved in interactions that are strongly regulated during EMT progression. We predict that interrupting these molecules and pathways will have an impact on EMT.

**Fig 6:**
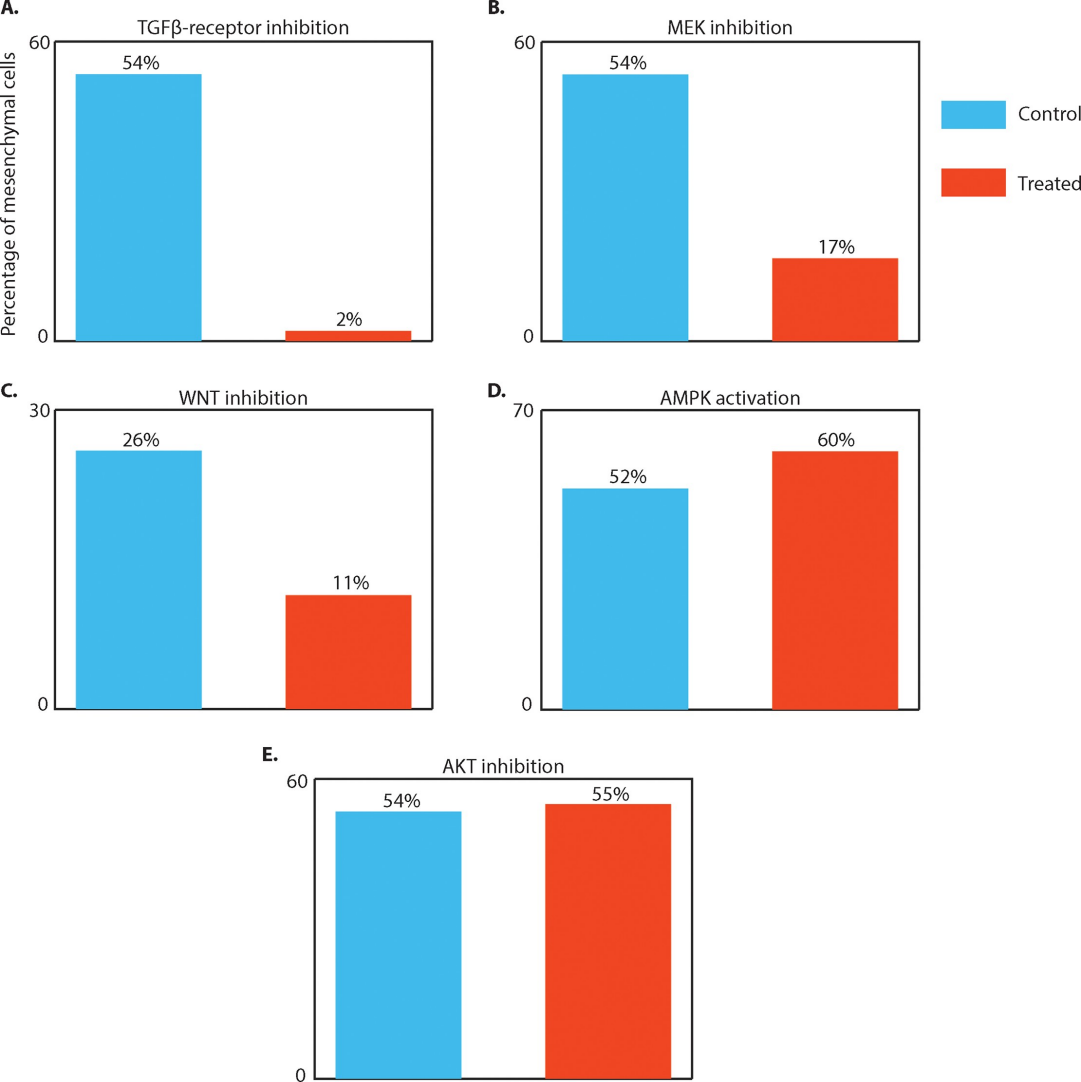
**Validation of critical edges for EMT.** (A)-(E) Bar plots showing the percentage of mesenchymal cells under control (TGFß stimulation) and under perturbation of the stated molecule for 5 days. The percentage values measure the impact of the perturbation on EMT. Manual gates were defined to identify mesenchymal cells (see Methods). (A) Inhibition of TGFß-receptor substantially reduces the fraction of cells completing the EMT transition from 54% under control to 2% following inhibition. (B) MEK inhibition also has a large impact on EMT, under which the fraction of mesenchymal cells drops to 17% from 54% under control. (C) WNT inhibition also causes the fraction of cells completing the transition to drop to 11% from 26% under control. (D) Activating AMPK on the other hand seems to slightly push cells into EMT as the fraction of mesenchymal cells increases from 52% to 60%. (E) AKT inhibition on the other hand has no impact on EMT, fraction of cells completing the transition is 55% compared to 54% under control.

To validate whether our driver edge predictions modulate EMT, we perturb these edges using drug inhibitions and activations. To determine the effect of the modulation on the EMT phenotype, we chronically inhibited/activated the respective molecules and pathways for 5 days while treating the Py2T cells with TGFß (see Methods). For comparison, cells were only treated with and without TGFß for the same time. As an additional negative control we used AKT inhibition as an example of a molecule that does not score high in the critical edge list (although typically associated with EMT in other systems). We then compare the percentage of cells that transitioned as measured by mass cytometry (Fig 6).

Inhibited molecules/pathways from our predicted critical edges include 1) SMAD2/3 pathway, 2) MEK/ERK/MAPK pathway, 3) ß-catenin/WNT pathway and 4) AMPK. All results shown in Fig 6 are supported by biological replicates (S7 Fig).

1. **SMAD2/3:** Upon TGFß stimulation, SMAD2/3 is phosphorylated by the TGFß-receptor [22], thus inhibition of the TGFß-receptor (SB431542) will abrogate SMAD phosphorylation. We find that inhibition of the TGFß-receptor causes the strongest impact on the progression. The fraction of cells that complete the transition drops to 2% under TGFß-receptor inhibition as compared to 54% in the control (Fig 6A).
2. **MEK1/2-ERK1/2**: Inhibition of MEK1/2 (PD318088) blocks the activity of the MAPK pathway (and therefore also the activity of ERK1/2 and P90RSK). Under the MEK1/2-inhibition, the fraction of cells that complete the transition drops to 17% (Fig 6B), less than a 1/3 of the cells that transitioned under control conditions, supporting its role in driving EMT [13].
3. **ß-catenin:** To probe WNT/ß-catenin pathway, we use the drug XAV-939, which is known to perturb WNT signaling and cause further ß-catenin degradation. Under this inhibition, the fraction of cells that complete the progression drops to 11% from 26% and 46% from 53% in control, respectively (Fig 6C).
4. **AMPK**: For AMPK, we tested an activator rather than an inhibitor, Phenformin. Activation of AMPK slightly increased the percentage of cells that underwent EMT to 60% compared to 52% in control (Fig 6D).

Additionally, we tested the AKT inhibitor (PHT427) as a negative control. AKT has been reported to be an important regulator in EMT for other cell lines such as human squamous carcinoma cells (SCC13 and SCC15) [23] and NMuMMG mammary epithelial cells [24]. Despite the AKT pathway being reported as prominent in the literature [17], we find edges involving AKT to be low in our ranking and indeed we empirically measure that AKT inhibition has little to no impact on EMT (Fig 6E, 55% of cells transition, compared to 54% on control). This result illustrates that EMT is not driven by the same edges across different systems.

In summary, 3D-DREMI successfully predicted important molecules and pathways involved during EMT, suggesting that the 3D DREMI analysis can be used to generate novel hypotheses on edges that are most relevant for a biological process of interest.

## Discussion

Here, we studied a cell-state transition system, and examined how regulatory relations are modulated during this transition. Specifically, we presented 3D-DREVI, 3D-DREMI and TIDES to visualize and quantify how the strength and shape of the relationship between two proteins change during a cell state transition. Importantly, we learned these dynamics from a single time-point of multidimensional single-cell data through a combination of pseudotime analysis and dependence-tracking along the resulting trajectory. Current high-throughput single-cell techniques offer high-dimensional snapshot measurements of thousands of cells, but typically lack dynamics. We utilized the variation in cell state, due to the variability and asynchrony in transition rates [25] to extract pseudo-temporal dynamics. A major assumption underlying our approach is that while the cells progress through EMT at different rates, they largely do so along a similar path. Therefore, we were able to map the process along a pseudotime dimension despite the inability to follow any single cell. Once cells were aligned along their position in a pseudotime trajectory, we tracked how relationships between molecules changed by formulating a dynamic model of edge strength. As such, our approach enables a dynamic view on molecular relationships in cell state transition and developmental processes. While several approaches have been proposed for studying interactions between proteins [10, 26], many of them have severe limitations that restrain their applicability in single-cell data. Our approach overcomes several of such limitations such as requiring prior knowledge of network topology, steady state assumptions, assuming continuity in protein concentration, computational inefficiency and the need to choose model parameters, thus standing out as a useful approach to study signaling in single cells.

We validated our methods using acute and chronic perturbations in the EMT system. Indeed, phases of higher dependency between proteins resulted in a larger impact upon perturbation. Moreover, we confirmed that the perturbation of these highly dynamic molecules, as predicted by our analysis, enabled the identification of nodes that halt EMT. Hence studying the dynamics can inform us of the key players involved in the transition and aid the selection of drugs that target key factors.

Differentiation (or trans-differentiation like in EMT) is essentially a process of gene and protein network rewiring and modulation. Thus, treating gene or protein networks as static fundamentally misses key aspects of this dynamic behavior. In our study we analyzed the epithelial-to-mesenchymal transition, which has important roles during development, wound healing, tissue fibrosis and cancer. However, our methods are generic, and can be utilized in any system that has a large number of cells distributed in a time-asynchronous way with respect to a process. Our method is useful in deriving time-dependent dynamics, and for identification of key proteins driving the transition.

The presented method makes one key assumption. It is only applicable if we have a continuous alignment of cells along a pseudotime. In this manuscript, we used Wanderlust [1] to align the cells along EMT onto a one-dimensional trajectory. However, biological processes can often involve bifurcations or multiple trajectories [2]. In such scenarios, our method can be easily adapted to study protein interaction along any particular branch or trajectory. On the other hand, complex tissues and disease systems can involve multiple cell-types that may not have overlapping sets of cells. In such cases, it is not possible to align all the cells along a trajectory. Thereby, the key assumption made by our method is violated making it inapplicable for such systems involving discrete subpopulations of cells.

Currently single-cell technologies are rapidly developing and enabling the measurement of more cells and molecules of interest. Single-cell RNA-sequencing [27, 28] provides a transcriptome-wide single-cell snapshot measurement and has enabled us to query complex biological systems under normal, diseased or complicated perturbations. The method presented in this manuscript can be extended to such higher dimensional data types, and allow for the study of gene-gene relationship in diverse settings. A challenge to this approach is that single-cell RNA sequencing (scRNA-seq) data tends to be sparse, typically capturing only about 10% of the molecules whereas DREMI and the higher dimensional versions formulated here require sufficient amounts of data to estimate density in the full dynamic range of molecules. Nevertheless, recently proposed imputation and data de-noising methods [29–32] could be used to accurately impute the data thus making our method more applicable. In addition, several computational tools have been developed that allow data-driven inference of developmental trajectories [2, 3] in complex tissues [33–35]. Given such advancements, our method can be readily adapted to scRNA-seq data with continuous progression of cells and thereby study gene-gene dynamics along a trajectory. For example, our approach could be applied to understanding the modulation in regulatory systems that govern malignant processes, opening up exciting possibilities. Finally, results in mass cytometry are a function of the used antibodies and changes in cell volume and cell cycle might influence results. Our antibodies used here were thoroughly validated and our findings, many of which reproduce known biology, underline the information content of the data.

## Methods

### Py2T cell culture and stimulation

Py2T cells were obtained from the laboratory of Gerhard Cristofori, University of Basel, Switzerland [13]. Cells were tested for mycoplasma contamination upon arrival and regularly during culturing and before being used for experiments. Cells were cultured at 37°C in DMEM (D5671, Sigma Aldrich), supplemented with 10% FBS, 2 mM L-glutamine, 100 U/ml penicillin, and 100 μg/ml streptomycin, at 5% CO_2._ For cell passaging, cells were incubated with TrypLE™ Select 10X (Life Technologies) in PBS in a 1:5 ratio (v/v) for 10 minutes at 37°C. For each experiment, cells were seeded at the density of 0.3 million cells per plate (100 mm diameter) and allowed to recover for 36 hours. After reaching 60% confluence, cells were either mock treated or treated with 4ng/ml TGFß (Human recombinant TGFß1, Cell Signaling Technologies) for 2, 3 and 4 days. Cell growth media and 4ng/ml TGFß treatment was renewed every day.

### Cell harvesting

For cell harvest, cells were washed two times with PBS and incubated with TrypLE™ Select 10X (Life Technologies) in PBS at a 1:5 ratio (v/v) for 10 minutes at 37°C. Following cell detachment, cells were cross-linked by addition of formaldehyde at a final concentration of 1.6% for 10 minutes at room temperature. Cross-linked cells were then centrifuged at 600 × *g* for 5 minutes at 4°C. After aspirating the supernatant, the cell pellet was re-suspended in -20°C methanol to a suspension of 1 million cells/ml and transferred to −80°C for long-term storage.

### Metal-labeled antibodies

Antibodies were obtained in carrier/protein free buffer and labeled with isotopically pure metals (Trace Sciences) using MaxPAR antibody conjugation kit (Fluidigm), according to the manufacturer’s standard protocol. After determining the percent yield by measurement of absorbance at 280 nm, the metal-labeled antibodies were diluted in Candor PBS Antibody Stabilization solution (Candor Bioscience GmbH) for long-term storage at 4°C. Antibodies used in this study are listed in S1 Table.

### Mass-tag cellular barcoding and antibody staining

Cell samples in methanol were washed three times with Cell Staining Media (CSM, PBS with 0.5% BSA, 0.02% NaN_3_) and once with PBS at 4°C. The cells were then re-suspended at 1 million cells/ml in PBS containing barcoding reagents (^102^Pd, ^104^Pd, ^105^Pd, ^106^Pd, ^108^Pd, ^110^Pd, ^113^In and ^115^In,) each at a final concentration of 100 nM. Cells and barcoding reagent were incubated for 30 minutes at room temperature. Barcoded cells were then washed three times with CSM, pooled and stained with the metal-conjugated antibody mix (S1 Table) at room temperature for 1 hour. Unbound antibodies were removed by washing cells three times with CSM and once with PBS. For cellular DNA staining, an iridium-containing intercalator (Fluidigm) was diluted to 250 nM in PBS containing 1.6% PFA and added to the cells at 4°C for overnight incubation. Before measurement, the intercalator solution was removed and cells were washed with CSM, PBS, and ddH_2_O. After the last washing step, cells were resuspended in MilliQ H_2_O to 1 million cells/ml and filtered through a 40-μm strainer.

### Mass cytometry analysis

EQ^TM^ Four Element Calibration Beads (Fluidigm) were added to the cell suspension in a 1:10 ratio (v/v). Samples were analyzed on a CyTOF1 (DVS Sciences). The manufacturer’s standard operation procedures were used for acquisition at a cell rate of ~300 cells per second as described in [14]. After the acquisition, all FCS files from the same barcoded sample were concatenated using the Cytobank concatenation tool (http://www.support.cytobank.org/hc/en-us/articles/206336147-FCS-file-concatenation-tool). Data were then normalized [36], and bead events were removed. Cell doublet removal and de-barcoding of cells into their corresponding wells was done using a doublet-free filtering scheme and single-cell deconvolution algorithm [37]. Subsequently, data was processed using Cytobank (http://www.cytobank.org/). Additional gating on the DNA channels (^191^Ir and ^193^Ir) was used to remove remaining doublets, debris and contaminating particulate.

### Immunofluorescence microscopy analysis

Cells were seeded on 12 mm glass coverslips in 24-well plates. After reaching 60% confluence, cells were treated with TGFß for 3 and 5 days. The cell growth media containing 4ng/ml TGFß was replenished once per day. All sample preparation steps were performed at room temperature. Cell samples were cross-linked with 4% paraformaldehyde in PBS for 20 min and permeabilized using 0.1% Triton X-100 in PBS for 3 min. After a blocking step with 0.5% BSA in PBS for 20 min, cell samples were incubated with the primary antibodies (E-Cadherin, Alexa Fluor® 647, 36/E-Cadherin, BD Biosciences; and Vimentin (D21H3) XP^®^ Cell Signaling Technologies) for 1.5 hours, and subsequently incubated for 1 hour with the appropriate fluorophore conjugated secondary antibodies (Alexa Fluor-488). Fluorophore-labeled antibodies were diluted in buffer containing 0.5% BSA in PBS. Nuclei were stained with Hoechst 33258 stain (Sigma Aldrich) diluted in PBS for 3 min. Coverslips were mounted in ProLong® Gold Antifade Mountant (Thermo Fisher Scientific) on microscope slides and imaged with a confocal microscope CLSM SP8 upright Leica. Images were acquired and analyzed using Imaris Software (Bitplane, Switzerland) and the acquisition was performed on the same day to prevent differences due to emission changes of the light sources. In addition, exposure times for a given marker were kept constant for the comparative analysis of each antibody.

### Time course experiment

Mock-treated and TGFß-treated cells were sampled for measurement after 2, 3 and 4 days. For each condition, three biological replicates were cultured, harvested and analyzed.

### Acute kinase inhibition

After chronic TGFß stimulation for 3 days, cells were treated with MEK1/2 (PD184352) small molecule inhibitors for 30 minutes at a concentration of 10μM and collected in two replicates.

### Chronic kinase perturbation

For chronic kinase perturbation, small molecule inhibitors (S4 Table) were applied to the cells at a concentration of 1 μM in parallel with TGFß or mock treatment. The small molecule inhibitor was applied once per day for 5 days, after media change and 10 minutes before TGFß stimulation, and collected in two replicates.

### Data preprocessing

All data were arcsinh transformed with a cofactor of 5 [14]. Any remaining debris or doublets were removed by gating on the DNA channels. For the time course and acute inhibition validation, the raw data was cleaned to remove cells that had spuriously high levels of certain signaling markers and transcription factors (pCREB, pSTAT5, pMEK1/2, pNFκß, Twist, Snail1 and Slug). An example between pCREB and pMEK1/2 is shown in Part A of S8 Fig. The effect is seen only in the markers whose metal antibodies have similar masses (S1 Table), hence indicating that the high correlation could be experimental noise. Further uninformative cells that had low levels of all markers were removed. For this, cells were clustered using Phenograph [38] on a set of phenotypic markers and transcription factors (E-cadherin, Vimentin, CD24, CD44, ß-Catenin, Snail1, Slug and Twist). The clusters of cells with low levels of markers were discarded thereafter (Part B of S8 Fig). The *junk* cells present in the data used for validation via acute inhibition (Fig 5 and S6 Fig) were also removed using Phenograph on the set of available phenotypic markers and transcription factors (E-cadherin, Vimentin, CD24, CD44, ß-catenin, Snail1 and Slug).

### Assessing cellular heterogeneity

We quantified the proportion of cells that complete the transition (Fig 1C and S1 and S2 Figs) by manually gating cells into various stages based on the expression levels of the canonical markers, E-cadherin and Vimentin. Cells with expression level of Vimentin < 2 were defined as epithelial cells, those with E-cadherin < 2.5 and Vimentin > 4 were defined as mesenchymal and rest of the cells as transitional. The same gates were used for all time-course data.

### Overview of computational methods to quantify edge dynamics

The computational methods developed in this paper are geared towards learning time-varying edge dynamics from static snapshot data. We study pairwise relationships as a function of time in cells undergoing the epithelial-to-mesenchymal transition. Studying such cell state dynamics from a single time point require computational techniques that can efficiently harness the rate of variability within large samples of cells to capture the transient dynamics.

We develop information theoretic techniques to study edge relationships as a function of pseudotime. These methods quantify the edge strength and describe time-varying edge shape. In particular, we develop:

1. *3D-DREVI (3D conditional Density Rescaled Visualization)* to visualize and characterize the relationship between a pair of molecules, *Y* and *Z*, along time *T*. For this, we compute the conditional density estimate $\hat{p}(Z|T,Y)$ to capture the dependency of *Z* on *T* and *Y* and use it visualize the average expression of *Z* given *Y* and *T*.
2. *3D-DREMI* (3D conditional Density Resampled Estimate of Mutual Information) to quantify the strength of relationship of *Z* on both *T* and *Y* by computing the differential entropy of *Z* when conditioned on *T* and *Y*.
3. *TIDES (Trajectory Interpolated DREMI Scores)* to quantify the relationship between two molecules continuously along time. This involves computing 2D-DREMI on fixed-time slices in the 3D space to derive the time-varying strength of the relationship.

First, we use Wanderlust [1] to align cells along a one-dimensional EMT-trajectory, which we call EMT-time. We treat EMT-time (*T*) as the x-variable and a pair of molecules *Y* and *Z* as the y and z-variables in order to compute 3D-DREVI, 3D-DREMI and TIDES. Underlying all our methods is the estimation of the joint density $\hat{p}(T,Y,Z)$, obtained using a fast heat-diffusion based kernel density estimate [16], which we extended to 3 dimensions. The methods are detailed as follows.

#### Kernel density estimation

Kernel Density Estimation (KDE) is a data-driven approach for learning the underlying probability density function [39]. Given a set of points in $(x_{1},x_{2},x_{3},\ldots ,x_{n})\in R$, a kernel density estimate for the distribution of the points is given by,

$$\hat{f}(x)=\frac{1}{n}\sum_{i=1}^{n}K_{h}(x-x_{i})$$

where, *K*_*h*_ is the kernel function. A popular choice of kernel is the Gaussian kernel, given by,

Kh(x)=1h2πe−x22h2

where, *h* is the bandwidth of the kernel. In higher dimensions, the kernel density estimate has the same form with points replaced by vectors.

#### Heat-equation based KDE

A standard method for computing a kernel density estimate amounts to evaluating a kernel function, *K*_*h*_, at every data point and summing the result. However, this method can be computationally challenging for large data sets. Instead, we use a method based on heat diffusion [16], which has previously been used successfully in single cell data sets [9] for 2D-KDE. The method estimates the underlying distribution by modeling it as the spreading of heat governed by the heat equation (with delta functions at the data points as the initial condition). The intuition is that the fundamental solution to the heat equation, in an infinite domain with Dirac delta function as the initial condition, is a Gaussian function. Mathematically, the solution to

$$\frac{\partial }{\partial t}\hat{f}(x,t)=\frac{1}{2}\frac{\partial^{2}}{\partial x^{2}}\hat{f}(x,t);\;\hat{f}(x,0)=\frac{1}{n}\sum_{i=1}^{n}\delta_{x_{i}}(x),\;x\in R$$

is given by,

f^(x,t)=∑i=1n12πte−(x−xi)22t,t>0.

For practical purposes, we have finitely many data points, so we rely on the finite domain solution to heat equation as an approximation of the kernel density estimate. We enforce Neumann boundary conditions (derivative of the probability density function is 0 at the boundaries), which preserves the total probability mass (initial amount of heat) inside the boundary. Given the initial condition and the boundary conditions, the solution to the heat equation can be written as [40],

$$\hat{f}(x,t)=\frac{1}{n}\sum_{i=1}^{n}\sum_{k=-\infty }^{\infty }e^{-k^{2}\pi t/2}cos(k\pi x)cos(k\pi x_{i}).$$

Extending these ideas to 3-dimensions, and under Neumann same boundary conditions, we obtain that the solution to

$$\begin{array}{l} \frac{\partial }{\partial t}\hat{f}(x,y,z,t)=\frac{1}{2}\left[\frac{\partial^{2}}{\partial x^{2}}\hat{f}(x,y,z,t)+\frac{\partial^{2}}{\partial y^{2}}\hat{f}(x,y,z,t)+\frac{\partial^{2}}{\partial z^{2}}\hat{f}(x,y,z,t)\right];\;\hat{f}(x,y,z,0)\\ \;=\frac{1}{n}\sum_{i=1}^{n}\delta_{x_{i}}(x)\delta_{y_{i}}(y)\delta_{z_{i}}(z),\;x,y,z\in R, \end{array}$$

is given by,

$$\begin{array}{l} \hat{f}(x,y,z,t)\\ =\frac{8}{n}\sum_{a,b,c=1}^{n}\sum_{k,l,m=-\infty }^{\infty }e^{-{(k}^{2}+l^{2}+m^{2})\pi t/2}cos(k\pi x)cos(k\pi x_{a})cos(l\pi y)cos(l\pi y_{b})cos(m\pi z)cos(m\pi z_{c}). \end{array}$$

The solution can be efficiently computed using a fast Fourier transform (FFT) [41]. This results in an estimate of the underlying probability density function. For 1- and 2-dimensions, the bandwidth $\left(\sqrt{t}\right)$ is obtained as a non-parametric solution to a fixed-point iteration [16, 41]. However, this method of obtaining bandwidth does not generalize beyond 2-dimensions [42] and becomes expensive to compute numerically. To generalize these ideas to higher dimensions, in this case 3-dimensions, we choose the bandwidth using Silverman’s rule of thumb [43],

hj=(45n)17σj,

where *n* is the number of points, *σ*_*j*_ is the standard deviation in *j*^*th*^ direction.

#### Algorithm

The algorithm starts by binning the data into a histogram. This is already a rough estimate of the underlying probability density. Although fast to construct, a histogram is not smooth, over-fits the data and depends heavily on the size of the bin. However, the strength of the presented algorithm lies in the fact that the resulting histogram is treated as delta functions on equally spaced points and this is used as the initial condition for solving the heat equation. This reduces the sample space from the original data size to the number of bins, hence achieving a considerable gain in speed. Then we transform the data into the frequency domain using the discrete cosine transform (DCT), which can be implemented using FFT, applied onto this initial condition. To use FFT we set the number of bins in the histogram to be set to certain power of 2, 2^7^ in this manuscript for 3D-KDE. This separates the signal present in the histogram into high frequency (noise) and low frequency (informative), thus allowing us to remove the noise and preserve meaningful information. The transform is then allowed to evolve for a time *t* (square of the bandwidth obtained using the rule of thumb), which is equivalent to multiplying by the exponential term ($e^{-k^{2}\pi^{2}t/2}$) in the equation above, which is equivalent to low-pass filtering of the DCT. The resultant is then inverted (inverse-DCT) to obtain a smooth kernel density estimate, see S9 Fig. The method extends naturally to higher dimensions. Computing kernel density estimates using heat diffusion can be performed in *O*(*n* + *m* log *m*) ~ *O*(*n*) for *n* ≫ *m*, where *n* is the number of data points, *m* is the number of bins. A sketch of the algorithm is as follows [41],

1. Construct a histogram
2. Transfer histogram into frequency domain via a discrete cosine transform
3. Evolve DCT (multiply DCT by $e^{-k^{2}\pi^{2}t/2}$, where *t* is the square of the bandwidth, *k* = 1,…,*m*, and *m* is the number of bins)
4. Inverse DCT for solution.

#### 3D-DREVI

As with 2D-DREVI [9], the joint high-dimensional density estimate can reveal areas of the state space that are densely and sparsely occupied by cells. However, as dimensionality increases, the sparsity of the data, relative to the state space, has increasingly larger impact. Already in 3 dimensions, the joint density of variables *p*(*X*,*Y*,*Z*) is often not good at revealing the underlying relationship between *X*,*Y* and *Z* because the majority of cells may be within a restricted portion of the dynamic range. Therefore, to accentuate the dependencies between molecules, we consider the conditional relationship of *Z* given *X* and *Y*, thus capturing the dependencies across the full dynamic range [9]. To compute conditional density *p*(*Z*|*X*,*Y*), we normalize the joint density by the conditioning variables *X* and *Y*. Since it is difficult to visualize a 3-dimensional conditional density, we instead visualize the conditional mean of *Z* given *X* and *Y*, resulting in a 2D surface (Fig 3C).

#### Computing 3D-DREVI

We begin by computing a 3D kernel density estimate $\hat{p}(X,Y,Z)$ on a cubic mesh grid {*x*_*i*_,*y*_*j*_,*z*_*k*_, 0 < *i*,*j*,*k* < *m*,*m* is the number of bins} using our heat equation based approach described above. Then each vector in the *z*-axis (corresponding to a fixed *X*- and *Y*-value, *X* = *x*_*i*_,*Y* = *y*_*j*_) is renormalized by the marginal density estimate of *X* = *x*_*i*_,*Y* = *y*_*j*_,

$$\hat{p}(z|x_{i},y_{j})=\frac{\hat{p}(x_{i},y_{j},z)}{\hat{p}(x_{i},y_{j})}=\frac{\hat{p}(x_{i},y_{j},z)}{\sum_{k}\hat{p}(x_{i},y_{j},z_{k})}.$$

We thus obtain an estimate for the underlying conditional density *p*(*Z*|*X*,*Y*) on the cubic mesh grid.

#### Visualizing 3D-DREVI

Computing $\hat{p}(Z|X,Y)$ results in a 3-dimensional array, where each entry represents the value of the density estimate at a particular vertex on the 3D-mesh grid, making it difficult to visualize what is essentially a solid cube. Instead, we visualize a surface through the conditional mean of *Z* given *X* and *Y*. This incidentally is often the area of the highest conditional density. The conditional mean can be computed as follows,

$$E(z|x_{i},y_{j})=\sum_{k}z_{k}\times \hat{p}(z_{k}|x_{i},y_{j}),$$

which results in a matrix where each entry corresponds to the average value of *Z* conditioned on the values of *X* and *Y*. This can be depicted as a surface plot on the *X* and *Y* mesh plane.

In this manuscript, we use 3D-DREVI to illustrate the relationship between two molecules along EMT-time (e.g. Fig 3D and 3E). We treat the Wanderlust derived EMT-time (*T*) as the *X* variable and the two molecules as *Y* and *Z* variables. We estimated the joint density at 2^7^ x 2^7^ x 2^7^ points (128 bins in each axis) and obtain the conditional mean of *Z* given *T* and *Y* as described above and render it as a red surface plot. Given a pair of molecules (*Y* and *Z*) and EMT-time, 50 cells from the right tail of the distribution of *Y* were discarded to obtain a well-populated dynamic range of *Y*, analogous to [9]. Finally, we remove wrinkles from the surface by smoothing the conditional mean using a linear sliding filter (of span 20 along both *T* and *Y* axes), using the *smooth* function in MATLAB.

#### 3D-DREMI

Once 3D-DREVI is computed, we compute 3D-DREMI, an extension of DREMI [9], to quantify the strength of *Z*′*s* dependency on both *X* and *Y*. Similarly to DREVI, we evaluate the strength of this dependency by re-weighing the contribution of each grid point uniformly thus taking the full dynamic range of the function into account [9].

#### Computing 3D-DREMI

Given three variables *X*, *Y* and *Z* (we typically assume that *X* and *Y* both influence *Z*), we quantify the dependence of *Z* on both *X* and *Y*. 3D-DREMI is defined as the mutual information on data that is sampled from the rescaled denoised-conditional density,

Rescale:p^(zk|xi,yj)=p^(zk|xi,yj)maxk(p^(zk|xi,yj)),

$$\mathrm{Denoise}\;\mathrm{by}\;\mathrm{setting}\;\hat{p}(z_{k}|x_{i},y_{j})<\varepsilon \;\mathrm{to}\;0\;\mathrm{for}\;\mathrm{all}\;i,j.$$

We measure the change in entropy of *Z* when conditioned on both *X* and *Y*, by computing the differential entropy between *Z* and *Z*|*X*,*Y*. That is, compute

$$I^{c}(Z|X,Y)=H^{c}(Z)-H^{c}(Z|X,Y),\;\mathrm{where},$$

$$H^{c}(Z)=-\sum_{i}\sum_{j}\sum_{k}\frac{\hat{p}(x_{i},y_{j},z_{k})}{\hat{p}(x_{i},y_{j})}\log\left(\hat{p}(z_{k})\right),\mathrm{and}$$

$$H^{c}(Z|X,Y)=-\sum_{i}\sum_{j}\sum_{k}\frac{\hat{p}(x_{i},y_{j},z_{k})}{\hat{p}(x_{i},y_{j})}\log\left(\hat{p}(z_{k}|y_{j},x_{i})\right),$$

where, *c* indicates that the mutual information and entropies are computed on the *c*onditional density.

This is a natural extension of 2D-DREMI as detailed in [9]. By treating EMT-time (*T*) as the *X-*variable, we can assess the strength of relationship between *Y* and *Z* throughout EMT-time. Pairs with high 3D-DREMI scores have a strong relationship with each other during the course of EMT-time. By ordering edges based on their 3D-DREMI score, we find candidate proteins that might be critical during EMT (S3 Table).

#### TIDES

3D-DREMI quantifies the relationship between two molecules throughout EMT-time. However, it does not provide information about the strength of the relationship at a given EMT-time. We developed a method based on 2D-DREMI to evaluate how a relationship changes continuously with EMT-time. We call this method TIDES for Trajectory Interpolated DREMI Scores.

#### Computing TIDES

We start with the rescaled conditional density estimate of *Z* given *T* and *Y*, where we consider EMT-time (*T*), as the *X-*variable. This 3-dimensional density estimate is projected onto a slice along the *Y*-*Z* plane, resulting in the conditional dependence of *Z* on *Y* for various fixed values of *T*. The projections are obtained by linearly interpolating the 3D density estimate onto a 2-dimensional slice, {(*t*_*i*_,*y*_*j*_,*z*_*k*_):0 < *j*,*k* < *m*, and *i* is a fixed value, *m* is the number of bins}, along *Y* and *Z* direction, for which we use the *interp3* function in MATLAB. The resulting conditional density estimate is denoised at *ε* = 0.9 to eliminate the technical noise from measurement [9],

$$\hat{p}(z_{k}|t_{i},y_{j})<\varepsilon \;\mathrm{to}\;0\;\mathrm{for}\;\mathrm{all}\;j,k\;\mathrm{and}\;\mathrm{fixed}\;i.$$

2D-DREMI computed on the slice quantifies the relationship at the fixed EMT-time *T*,

$$I^{c}(Z|Y)=H^{c}(Z)-H^{c}(Z|Y),\;\mathrm{where}$$

$$H^{c}(Z)=-\sum_{i}\sum_{j}\frac{\hat{p}(y_{i},z_{j})}{\hat{p}(y_{i})}\log\left(\hat{p}(z_{j})\right),\;\mathrm{and}$$

$$H^{c}(Z|Y)=-\sum_{i}\sum_{j}\frac{\hat{p}(y_{i},z_{j})}{\hat{p}(y_{i})}\log\left(\hat{p}(z_{j}|y_{i})\right),$$

where, *c* indicates that the mutual information and entropies are computed on the *c*onditional density.

#### Visualizing TIDES

Repeatedly computing TIDES for several values along EMT-time allows continuous tracking of edge strength during the EMT transition, resulting in a TIDES curve. We compute TIDES values at 256 locations along EMT-time, twice the number of bins used to estimate the density. Once computed, the TIDES curves were smoothed using a Gaussian filter. For this, a Gaussian centered at each value of EMT-time (on which TIDES is computed) is used to estimate the weighted average at each location. Averaging the values results in a smooth TIDES curve. The weights are determined as follows,

$$K_{jl}=\frac{1}{\sqrt{2\pi }\sigma^{2}}exp\;\left(-\frac{{\left(\tau_{j}-\tau_{l}\right)}^{2}}{\sigma^{2}}\right),$$

where, *τ*_*j*_ is the TIDES value at EMT-time *j*, *τ*_*l*_ is the mean TIDES value in the bin *l* and *σ* is the bandwidth of the Gaussian chosen using Silverman’s rule of thumb [43]. The weighted average is then calculated as,

$$T_{l}=\sum_{j=1}^{256}K_{jl}*\tau_{j}.$$

### Deriving wanderlust pseudotime

We used Wanderlust [1], a graph-based trajectory detection algorithm, to align the cells onto a one-dimensional axis representing the transition of cells from epithelial to mesenchymal phenotype. We call the resulting pseudotime axis as EMT-time. EMT-time is normalized to 0–1, where epithelial cells are near 0 and mesenchymal cells near 1. We compute EMT-time by running Wanderlust on a set of phenotypic markers and transcription factors: E-cadherin, Vimentin, CD44, ß-catenin, Snail1, and Slug. A shared nearest neighbor graph was constructed using *K* = 60 nearest neighbors and shared nearest neighbor (snn) = *K*/3 = 20. The parameter *l* which is used to choose *l out of K* neighbors (to avoid short circuits) was set to K/5 = 12. The constructed trajectory is robust to these parameters (S10 Fig). The *start* point was set to the set of the cell with low E-cadherin and high Vimentin. In particular, the cell with maximum expression of Vimentin from the set of cells whose expression of E-cadherin < 1.5 and Vimentin > 4.5 was chosen as the *start* point. The resulting trajectory was then inverted. The number of graphs (over which the result of the algorithm is averaged) was set to 5.

Once generated, we study the expression of various markers along EMT-time (e.g. Fig 2B and 2C, Parts A-C of S3 Fig). The marker trends were generated by first partitioning EMT-time into 256 equally spaced bins, by dividing the range of the Wanderlust score by 256. Then the weighted average of the marker using a Gaussian filter centered at the bin is computed, as detailed in [15]. The weights are calculated as follows,

$$K_{jl}=\frac{1}{\sqrt{2\pi }\sigma^{2}}exp\;\left(-\frac{{\left(m_{j}-{\bar{m}}_{l}\right)}^{2}}{\sigma^{2}}\right),$$

where, *m*_*j*_ is the marker expression of cell *j*, ${\bar{m}}_{l}$ is the mean marker expression value in the bin *l* and *σ* is the bandwidth of the Gaussian chosen using Silverman’s rule of thumb [43]. The weighted average is then calculated as,

$$E_{l}=\sum_{j=1}^{N}K_{jl}*m_{j},\mathrm{where}\;N\;\mathrm{is}\;\mathrm{the}\;\mathrm{total}\;\mathrm{number}\;\mathrm{of}\;\mathrm{cells}.$$

#### Consistency of marker trends along EMT-time across replicates

We demonstrate that the marker trends are consistent across replicates (Part D of S3 Fig). For a given day, the expression of a marker along EMT-time from one replicate was cross-correlated with the expression of the same marker along EMT-time from another replicate. This was repeated for all the markers and average correlation was computed. The computation was done for all 3 replicates from Day 2, 3 and 4. The markers used were: pCREB, pSTAT5, pFAK, pMEK1/2, Twist, cmyc, Snail1, pNFκB, pP38, pAMPK, pAKT, pERK1/2, Slug, Cyclinb1, CAIX, pGSK3ß, pSMAD1/5, CD44, Vimentin, pSMAD2/3, ß-catenin, pMARCK, CD24, pPLCγ2, pPH3, pS6, E-cadherin, cleaved caspase 3 (ccasp), pSTAT3, pRb, Survivin.

#### Consistency of marker trends along EMT-time across days

We also find that the marker trends are consistent across days (Part E of S3 Fig). The expression of a marker along EMT-time on replicate 1 of Day 2 was correlated with the expression of the same marker along EMT-time on replicate 1 of Day 3. This was repeated for all the markers and average correlation was computed. The same is done to compare replicate 1 of Day 2 against Day 4 and replicate 1 of Day 3 against Day 4. The final result is rendered as a heat-map. Similar heat-maps were generated for replicates 2 and 3. The markers used were: pCREB, pSTAT5, pFAK, pMEK1/2, Twist, c-myc, Snail1, pNFκB, pP38, pAMPK, pAKT, pERK1/2, Slug, Cyclinb1, CAIX, pGSK3ß, pSMAD1/5, CD44, Vimentin, pSMAD2/3, ß-catenin, pMARCKS, CD24, pPLCγ2, pPH3, pS6, E-cadherin, ccasp, pSTAT3, pRb, Survivin.

#### Consistency of signaling controlled for EMT-time

We demonstrate that signaling is similar across days when controlled for EMT-time (Fig 3A and 3B, Part A of S4 Fig). Cells from days 2, 3 and 4 were divided into four groups based on EMT-time: cells with EMT-time < 0.25 (Group-1), between 0.25 and 0.5 (Group-2), between 0.5 and 0.75 (Group-3) and greater than 0.75 (Group-4). DREMI on all pairs of signaling molecules in each of the groups was computed. Then the DREMI scores from a group was correlated with the DREMI scores from the same group on a different day, and the final result is rendered as a heat map. We used signaling markers to perform this analysis, since were are interested in whether signaling pattern is maintained between days. The markers used are: pCREB, pSTAT5, pFAK, pMEK1/2, Twist, c-myc, Snail1, pNFκB, pP38, pAMPK, pAKT, pERK1/2, Slug, Cyclinb1, pGSK3ß, pSMAD1/5, pSMAD2/3, ß-catenin, CAIX, pMARCK, pPLCγ2, pPH3, pS6, pSTAT3 and pRb.

### Validating short-term drug inhibition

We used short-term drug inhibition to validate rewiring suggested by TIDES. For a given pair of molecules *Y* and *Z*, TIDES (*Y* → *Z*) quantifies the strength of the statistical relationship between *Y* and *Z* continuously along EMT-time (*T*). We assume that inhibiting *Y* or some molecule immediately upstream of *Y* should have higher impact on *Z* in the region where the TIDES score is high, and analogously the impact should lower in regions of lower TIDES scores. To validate TIDES, we compute TIDES curve of *Y* − *Z* and cross-correlate it with the impact curve of *Z*, defined as the expression of *Z* under control minus the expression of *Z* under treatment. A high correlation would indicate that TIDES correctly predicts the regions of strong/weak relationship. For analysis, both the curves were normalized to 0 to 1 and cross-correlated on the EMT-time axis. The shift which provided the maximum cross-correlation was chosen and the Pearson correlation value was reported.

### Validating long-term drug inhibition

We defined manual gates, based on the levels of E-cadherin and Vimentin, for computing the fraction of mesenchymal cells to validate the impact of long-term drug perturbation on EMT (S7 Fig). The gates used are: (1) TGFß-receptor, MEK1/2 and AKT inhibition: E-cadherin < 3, vimentin > 4. (2) AMPK-perturbation: E-cadherin < 3 and Vimentin > 3.5. (3) WNT-inhibition: E-cadherin < 3 and Vimentin > 4 (Replicate 1), and E-cadherin < 3.5 and Vimentin > 4 (Replicate 2). Our predictions are validated across replicates.

### Runtime analysis

We performed runtime analysis of our methods. We first assessed how our method scales with size of the data. Since heat-diffusion based kernel density estimation starts off by computing the histogram of the data (S9 Fig), we fixed the number of bins to 128. For randomly generated data sets from uniform distribution, with sample size ranging from 5000 to 50000 (3 features for each data-point), the heat-diffusion based method computes KDE within 1 second, Part A of S11 Fig. The runtime is uniform across a range of data size because the algorithm is less dependent on the data size and more on bin size, which was kept constant here. Second, we studied the run time of our method against the number of bins in the initial histogram. We fixed the size of the data set to 5000 points (each with 3 features) and altered the number of bins in the initial construction of the histogram. For up to 256 bins in each direction (density estimated at 2^24^ points), the heat-diffusion based method computes KDE within 10 seconds (Part B of S11 Fig). We compared the runtime of our method to an alternative approach [44]. We used the code available at http://www.ics.uci.edu/~ihler/code/kde.html. As shown in Part C of S11 Fig, our method scales better than the alternative against the number of bins of histogram. Using the heat-diffusion based KDE, 3D-DREVI and 3D-DREMI can be computed within 25 seconds for 128 bins (Parts D and E of S11 Fig). Similarly, TIDES can be computed in less than 5 minutes (Part F of S11 Fig). For all of these experiments, since the method depends mostly on the number of bins for the histogram, only an example pair of edges (pS6—pGSK3ß) along EMT-time was chosen for three replicates from Day 3 (unless stated otherwise) and the average runtime was computed.

### Time complexity

Given *N* data points and *B* bins, 3D-KDE first computes a 3D-histogram (of size *B* × *B* × *B*) of the data, which scales as 3*NB*~*O*(*NB*) (S9 Fig for an illustration of the algorithm in 1D). Second, it computes the discrete cosine transform of the histogram using Fast Fourier Transform, which scales as *O*(*B*^3^log(*B*)) (for this we require *B* to be some power of 2). The result is then multiplied element-wise by the exponential term followed by an element wise sum, which costs *O*(*B*^3^). This is followed by inverse discrete cosine transform, which again costs *O*(*B*^3^log(*B*)). Overall, the algorithm’s time complexity is *O*(*NB* + *B*^3^log(*B*)).

### Performance analysis

In order to assess how the presented algorithm performs as a function of the number of bins, we first consider the case when the underlying probability density is known. We generate a 3-dimensional Gaussian distribution with mean 0.5 and variance 0.3 in each of the 3 dimensions. We then randomly sample 5000 points from this distribution and apply our method to approximate the underlying density function. Since we are estimating a 3-dimensional density function, we obtain a density estimate at nbins x nbins x nbins number of points. We use the sum of absolute difference between the ground truth distribution values and the estimated values at each of these points normalized by the number of estimated points (nbins^3^, that is nbins raised to power 3) as a measure of error. As shown in Part A of S12 Fig, our choice of 128 bins in each of X, Y and Z direction is a stable choice.

Second, to consider the effect of number of bins on our data, we compute the TIDES score for 100 randomly chosen pairs of proteins from Day 3 Replicate 1 data for various values of number of bins. The TIDES score are then correlated against each other and averaged over the 100 pairs of proteins. The final correlation matrix is shown in Part B of S12 Fig. The results obtained with smaller number of bins are inconsistent with results obtained with higher number of bins, while results obtained using larger number of bins are more consistent with each other. This suggests that there is not much improvement in the efficacy of the method on increasing number of bins from 128 to 256, which supports our choice of 128 bins.

### Comparison against other kernel choices

The formalism we employ here to construct Kernel Density Estimation by equating the underlying probability density function of some given continuous data as a solution to the heat-equation with initial condition the data itself, works only for Gaussian kernels. The choice of Gaussian kernel is motivated by our previous work [9]. However, in the present context we compare our proposed method against alternate kernels (linear and cosine). The linear kernel is given by,

$$K_{l}(x;h)=\left(1-\frac{\left|x\right|}{h}\right)\;I\;(\left|x|<h\right),$$

where *h* is the bandwidth and I is the indicator function (it is 1 if the argument, |*x*| < *h*, is true, else it is 0). Employing this in 3-dimensions, the resulting estimated probability density function is given by [45],

$${\hat{f}}_{l}(x,y,z;h_{x},h_{y},h_{z})=\frac{1}{n}\sum_{i=1}^{n}K_{l}(x-x_{i};h_{x})\;K_{l}(y-y_{i};h_{y})\;K_{l}(z-z_{i};h_{z}),$$

where (*x*_*i*_,*y*_*i*_,*z*_*i*_) are the given input data and *h*_*x*_,*h*_*y*_,*h*_*z*_ are the bandwidths in each of *x*,*y* and *z* directions. Similarly, the cosine kernel is given by,

$$K_{c}(x;h)=\frac{\pi }{4}\cos\left(\frac{\pi }{2}\frac{\left|x\right|}{h}\right)\;I\;(|x|<h),$$

where *h* is the bandwidth and I is the indicator function (it is 1 if the argument, |*x*| < *h*, is true, else it is 0). The estimated probability density function is given by [45], ${\hat{f}}_{c}(x,y,z;h_{x},h_{y},h_{z})=\frac{1}{n}\sum_{i=1}^{n}K_{c}(x-x_{i};h_{x})\;K_{c}(y-y_{i};h_{y})\;K_{c}(z-z_{i};h_{z})$. To evaluate the effectiveness of each of these kernels, we consider the 3D-DREVI plots generated due to each of these kernels (see Parts E and F of S9 Fig). As we can see, linear and cosine kernels lead to visually inferior results in terms of smoothness of the resulting DREVI plots. This is indicative that linear and cosine kernels are likely over fitting our data.

To quantitatively assess the effectiveness of our proposed choice of kernel, we compute robustness of the kernels to various sub samplings of the data set, and compare it against alternate kernels. For each kernel, we consider the density estimated on the full data set as the ground truth. Then we randomly subsample cells without replacement from the data to various sizes (e.g. to 90% of the original size) and re-estimate the underlying density in the same set of points as on the full data set. This allows us to directly compare the estimated densities. In particular, we compute the L1 norm of the difference between the estimated densities (ground truth computed in the original data and density estimated in subsampled data) and use it as a metric of robustness of the choice of kernel. Therefore, if *D*_*k*_ is the density estimated using kernel *k* in the original data, which serves as the ground truth for that kernel, and ${\hat{D}}_{k,sub}$ is the density estimated using kernel *k* in the subsampled data, then we define the error as $\sum \left|D_{k}-{\hat{D}}_{k,sub}\right|$. We report results for two example pairs of proteins in Parts G and H of S9 Fig. As we can see, the Gaussian kernel is much more robust than linear and cosine kernel.

### Data and software availability

All data and the software will be available at https://github.com/dpeerlab/tides.

## Supporting information

S1 Fig TGFβ treatment reproducibly induces EMT: (A-B) Contour plots of Vimentin and E-cadherin after 2–4 days of TGFβ stimulation; biological replicates for main Fig 1C. Replicates confirm a shift in density of cells from epithelial to mesenchymal phenotype with time and illustrate a continuum of cells in transition on days 2–4.
(TIFF)

S2 Fig EMT characteristics are consistent across replicates: (A) Scatterplot where each point is a cell treated with TGFβ for 3 days. The cells are divided into three distinct categories: Epithelial, Transitional and Mesenchymal (see Methods). (B)-(E) A distribution of marker levels is shown for the three categories. Expression of E-cadherin (B) and CD24 (C) is high in epithelial cells, decreases in transitional cells, and is much lower in mesenchymal cells, consistently across replicates. Expression of Vimentin (D) and CD44 (E) is low in epithelial cells, increases in the transitional cells, and is higher in the mesenchymal cells, consistently across replicates.
(TIFF)

S3 Fig A spectrum of marker trends along EMT-time are seen consistently across replicates: (A)-(C) Plots show the expression of various markers along Wanderlust generated EMT-time in the cells treated with TGFβ on Day 2, 3 and 4 respectively. Smoothing was performed by a sliding-window Gaussian filter. The shaded region around each curve indicates one standard deviation across replicates indicating consistency. (D) Plot showing the average cross-correlation of marker expression along EMT-time across replicates. For a given marker, the expression along EMT-time is cross-correlated across replicates. The average correlation over the set of markers is rendered as a heat map. (E) Average cross-correlation of marker expression along EMT-time is similar across the different days within each replicate.
(TIFF)

S4 Fig Signaling relationships along EMT-time in replicates: (A) TGFβ-treated cells from Days 2, 3 and 4 are binned into four groups along EMT-time. DREMI score between all pairs of signaling molecules is computed in each group. Heat map shows the correlation of the DREMI scores for each group across days. Average correlation is 0.68 (Replicate-2) and 0.73 (Replicate-3). (B) Dynamics of the relationship between pGSK3β and Snail1, similar to main Fig 3D across biological replicates. 3D-DREVI depicts the typical expression of Snail1 conditioned on pGSK3β and EMT-time. The modulation in the relationship is visualized by the 2D-DREVI slices along EMT-time and quantified the TIDES curve (purple curve) shown along the z-axis. (C) Dynamics of the relationship between pPLCγ2 and pMEK1/2 similar to Fig 3E across biological replicates.
(TIFF)

S5 Fig Information transfer during EMT across transcription factors: Average TIDES curve of the relationship between several molecules (pCREB, pSTAT5, pFAK, pMEK1/2, pNFκB, pP38, pAMPK, pAKT, pERK1/2, pGSK3β, pSMAD1/5, pSMAD2/3, β-catenin, CAH IV, pMARCK, pPLCγ2, pS6, pSTAT3) and Snail1 (B) and Twist (C), across three replicates for Day 3. Similar to Slug in main Fig 4, the curves start rising steadily at near EMT-time ~ 0.25, and peak near EMT-time ~ 0.75.
(TIFF)

S6 Fig Validation of TIDES via short-term drug inhibition for direct and indirect edges in replicates: (A) Cross-correlation of TIDES curve between pMEK1/2-pP90RSK with the impact curve of pP90RSK results in a high correlation. This is a biological replicate of main Fig 5A. (B) Cross-correlation of TIDES curve between pMEK1/2-pP90RSK with the expression level of pP90RSK under control. Lower correlation than in (A) indicates that TIDES does not trivially follow the levels of pP90RSK. The curves end at EMT-time ~0.5 as the control does not contain sufficient cells in the mesenchymal state. (C) Biological replicate of Fig 5B; cross-correlating TIDES curve between pMEK1/2-pERK1/2 with the impact curve of pERK1/2 results in a high correlation. (D)-(E) Cross-correlation of pERK1/2-pP90RSK TIDES curve and pP90RSK impact curve under MEK-inhibition is 0.84 and 0.80 across two replicates.
(TIFF)

S7 Fig Validation of critical edges for EMT via long-term drug inhibition in replicates: (A)-(E) Shown are contour plots of cells treated with TGFβ (Control) and with TGFβ plus a chronic drug perturbation of the stated molecule for 5 Days, across biological replicates. Results of replicate 1 were shown as bar plots in Fig 6. Inhibition of TGFβ-receptor (A), MEK (B) and WNT (C) cause a substantial decrease in the fraction of cells that complete transition, while activation of AMPK (D) increases the proportion of cells that complete transition. AKT (E) on the other hand does not seem to impact the transition.
(TIFF)

S8 Fig Data clean-up: (A). Scatterplot showing the relationship between pCREB and pMEK1/2 on Day 3 (shown is replicate 1). A spurious relationship between pCREB and pMEK1/2 at high pCREB values is seen. These events were manually gated out from time course and acute inhibition validation data sets. (B) Shown are heat maps of the expression of markers on various clusters obtained using Phenograph [46] on a set of phenotypic markers and transcription factors. The data shown is from Day 3 (replicate 1). The cells comprising the clusters with low expression of markers (such events are found in most mass cytometry experiments) were removed (indicated by red rectangles) from further analysis.
(TIFF)

S9 Fig Computing Kernel Density Estimate: (Α) Plot shows histogram of a randomly chosen marker on Day 3. Constructing the histogram of the data is the first step in computing kernel density estimate. The histogram represents the initial condition for solving the heat equation. (Β) The histogram is then transformed into frequency domain by the Discrete Cosine Transform (DCT). (C) A low-pass filter smooths the DCT by removing the noisy parts. This is obtained by multiplying the DCT by an exponentially decaying term (exp(-k^2^π^2^t/2), see Methods) or in other words, evolving the initial condition in time. (D) The smooth density estimate is derived by inverting the smoothed-DCT. (E) Plot shows 3D-DREVI of pERK1/2 -> pS6 along EMT-time for various choices of kernel (Gaussian, linear and cosine) used in the estimation of underlying 3D-density. (F) Same as (E) for pERK1/2 -> Snail1 along EMT-time. (G) Plot shows robustness of each of the kernels against various sub samplings of the data. The density estimated using the full data is taken as the ground truth and the error is defined as the sum of the absolute difference between density estimated in various subsamples of the data against the ground truth. The solid line indicates the mean and the error bars indicate 1-standard deviation across 20 repetition of subsampling. The result is shown for pERK1/2 -> pS6 along EMT-time using Day 3 Replicate 1 data. (H) Same as (G) for pERK1/2 -> Snail1 along EMT-time (see Methods).
(TIFF)

S10 Fig Robustness of Wanderlust generated EMT-time: (A) Heat-map shows the correlation between trajectories generated for various values of K-nearest neighbors. The shared nearest neighbor (snn) parameter was fixed at 20, while the *l* parameter (to avoid short circuits in the graph) was fixed at 12. (B) Heat-map shows the correlation between trajectories generated for various values of *l* parameter. K was fixed at 60 and snn was fixed at 20. (C) Heat-map shows the correlation between trajectories generated for various values of snn. K was fixed at 60 and *l* was fixed at 12. The results shown are for data on Day 3 (replicate 1), and holds true for all of our data.
(TIFF)

S11 Fig Runtime Analysis. (A) Runtime for computing heat-diffusion based KDE against data size. The data is uniformly generated with 3 features. The number of bins used to construct the initial histogram was fixed to 128 in each of the three directions. The green line shows the mean while shaded region shows one standard deviation for 100 iterations. (B) Runtime against the log of number of histogram bins (in each of the three directions) for 5000 uniformly generated points. Computing density estimates on 2^24^ points takes less than 10 seconds. (C) Runtime comparison of our method against an alternate (based on computational geometry) [47] for computing three-dimensional KDE. The shaded region shows the standard deviation across three replicates from the time-course data Day 3 for an edge (pS6-pGSK3β) and EMT-time. Our method can compute 3D density estimates at 2^24^ points in less than 10 seconds while the alternative takes more than 10 minutes. (D)-(F) Plots show runtime of heat-diffusion based method against log of number of bins in computing 3D-DREVI, 3D-DREMI and TIDES respectively. The shaded region shows the standard deviation in runtime across three replicates of data from Day 3 for the edge used in (C), and the middle line shows the mean runtime.
(TIFF)

S12 Fig **P**erformance Analysis: (A) Performance analysis of the presented method against number of bins. The plot shows reconstruction error against number of bins on a simulated data. The underlying distribution is a 3-dimensional Gaussian distribution with mean 0.5 and variance 0.3 in each of X, Y and Z directions. The reconstruction error is defined as the sum of absolute difference between the ground truth and the estimated density (using our method) for various number of bins normalized by the number of points density is estimated at (nbins x nbins x nbins). The density was estimated using 5000 randomly sampled points from the underlying distribution. The solid line shows the mean and the error bars show 1-standard deviation across 20 random sampling of the data points. (B) Plot shows the correlation in TIDES scores computed using various number of bins and averaged over a set of 100 randomly chosen pairs of proteins from Day3 Replicate 1 data.
(TIFF)

S1 Table List of molecules and antibodies used for all mass cytometry analysis except the acute inhibition experiment. (DOCX)

S2 Table List of molecules used to construct wanderlust pseudo-time. (DOCX)

S3 Table List of small molecules used for chronic perturbation. (DOCX)

S4 Table List of small molecules used for chronic perturbation. (DOCX)
