# Supplementary figures and images for: Learning time-varying information flow from single-cell epithelial to mesenchymal transition data
Source: PLoS One. 2018 Oct 29;13(10):e0203389. doi: 10.1371/journal.pone.0203389 (PMC6205587; doi:10.1371/journal.pone.0203389)

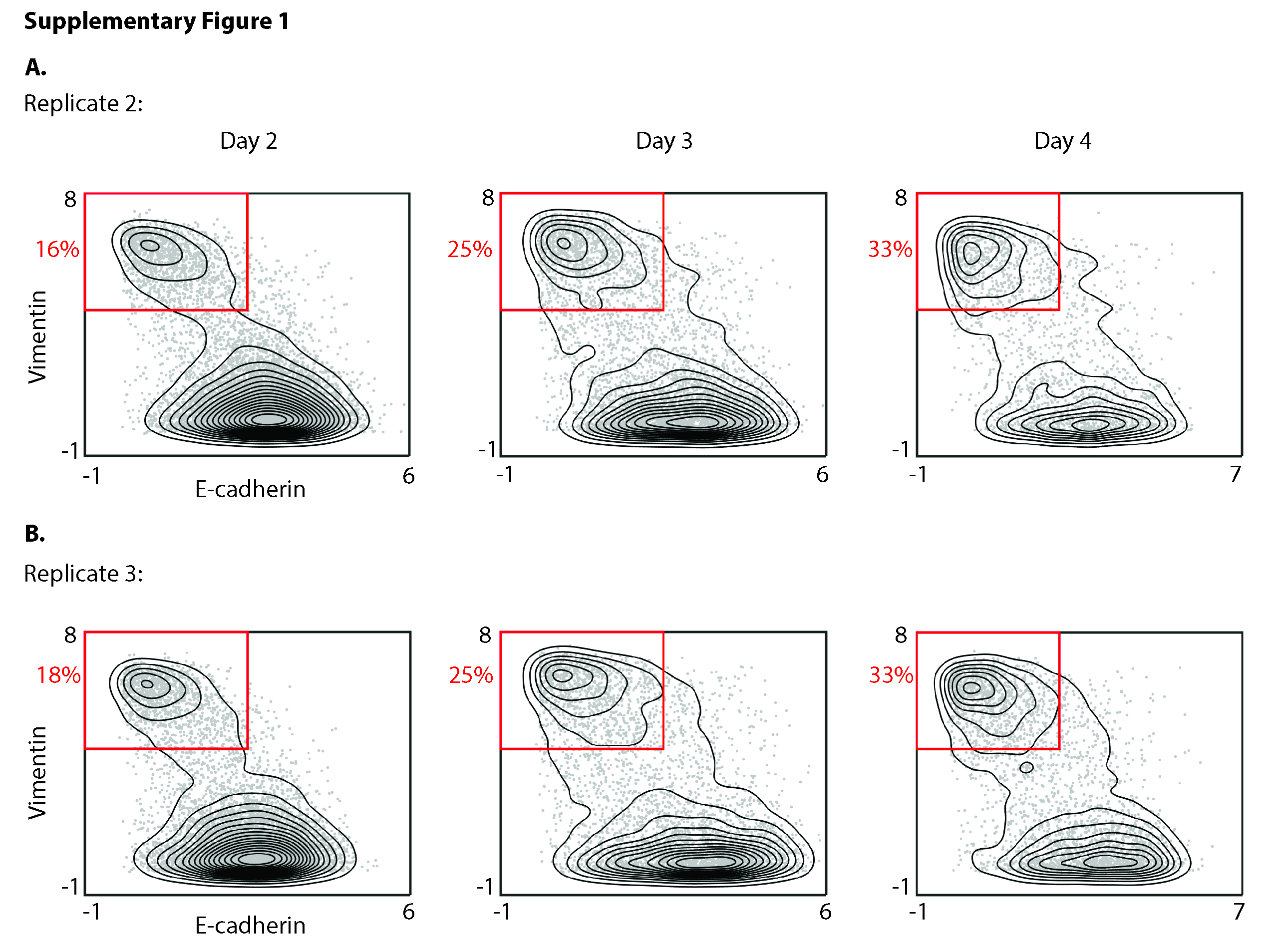

Supplement: S1 Fig — TGFβ treatment reproducibly induces EMT: (A-B) Contour plots of Vimentin and E-cadherin after 2–4 days of TGFβ stimulation; biological replicates for main Fig 1C. Replicates confirm a shift in density of cells from epithelial to mesenchymal phenotype with time and illustrate a continuum of cells in transition on days 2–4. (TIFF) [file pone.0203389.s001.tiff]

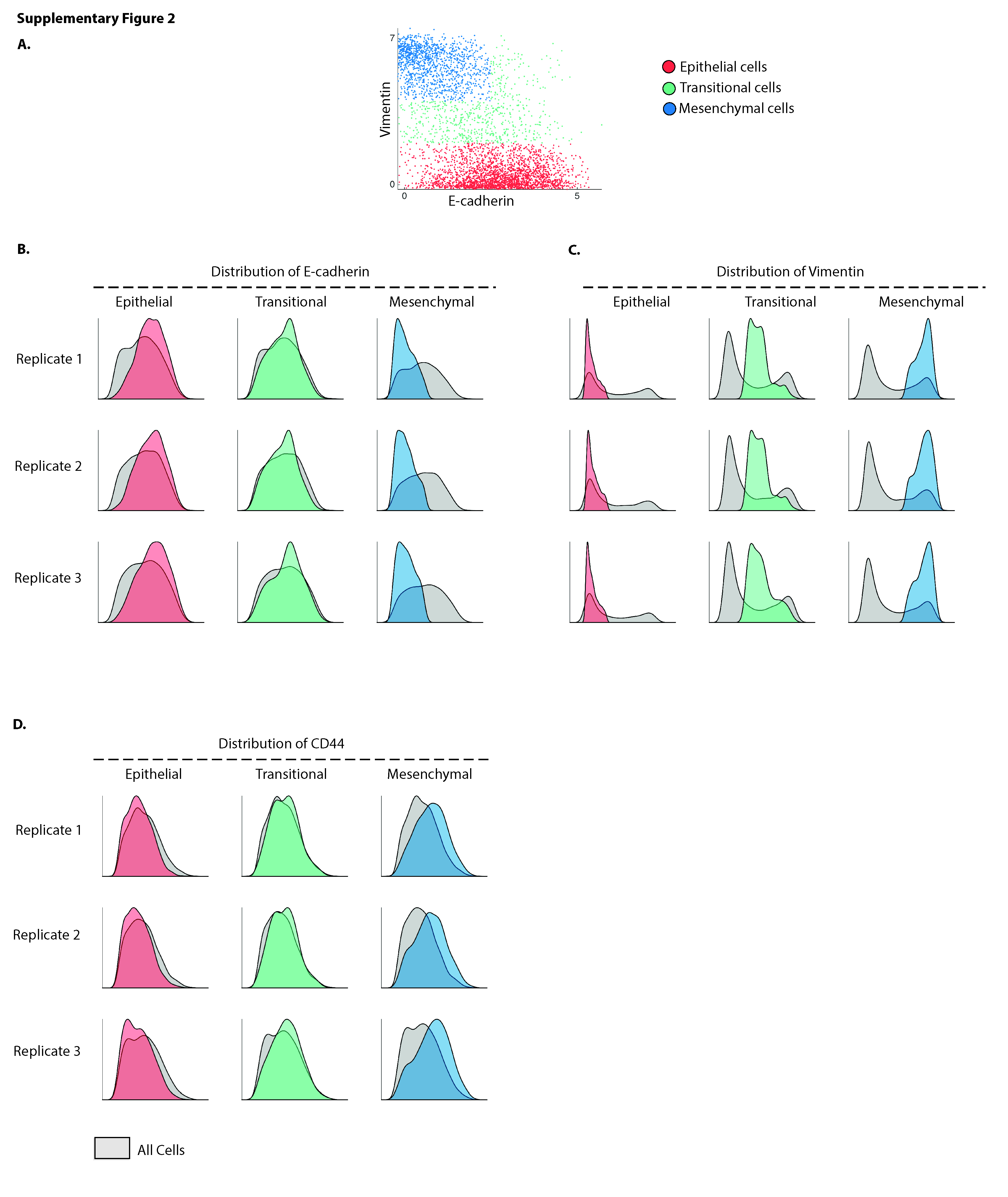

Supplement: S2 Fig — EMT characteristics are consistent across replicates: (A) Scatterplot where each point is a cell treated with TGFβ for 3 days. The cells are divided into three distinct categories: Epithelial, Transitional and Mesenchymal (see Methods). (B)-(E) A distribution of marker levels is shown for the three categories. Expression of E-cadherin (B) and CD24 (C) is high in epithelial cells, decreases in transitional cells, and is much lower in mesenchymal cells, consistently across replicates. Expression of Vimentin (D) and CD44 (E) is low in epithelial cells, increases in the transitional cells, and is higher in the mesenchymal cells, consistently across replicates. (TIFF) [file pone.0203389.s002.tiff]

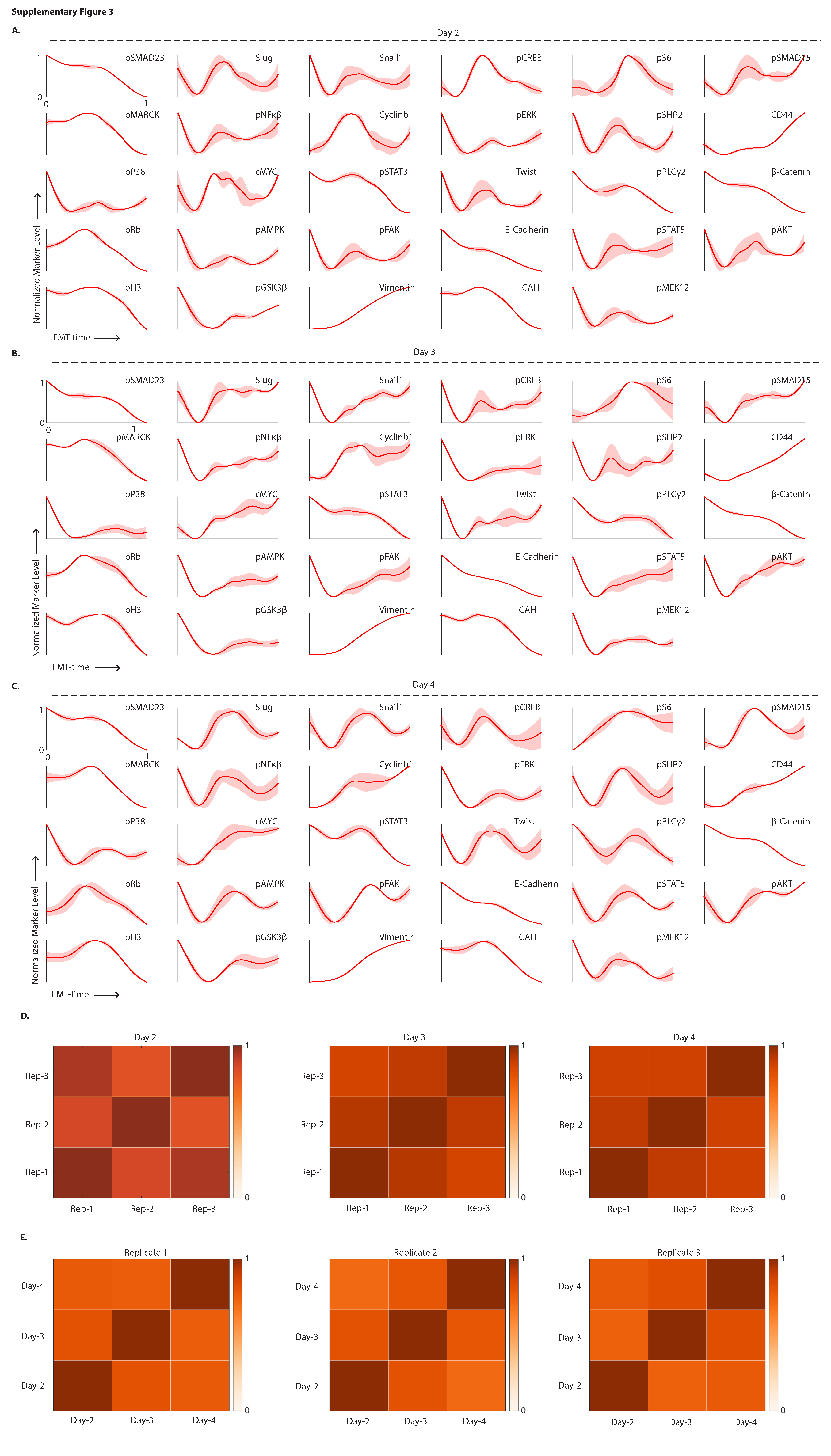

Supplement: S3 Fig — A spectrum of marker trends along EMT-time are seen consistently across replicates: (A)-(C) Plots show the expression of various markers along Wanderlust generated EMT-time in the cells treated with TGFβ on Day 2, 3 and 4 respectively. Smoothing was performed by a sliding-window Gaussian filter. The shaded region around each curve indicates one standard deviation across replicates indicating consistency. (D) Plot showing the average cross-correlation of marker expression along EMT-time across replicates. For a given marker, the expression along EMT-time is cross-correlated across replicates. The average correlation over the set of markers is rendered as a heat map. (E) Average cross-correlation of marker expression along EMT-time is similar across the different days within each replicate. (TIFF) [file pone.0203389.s003.tiff]

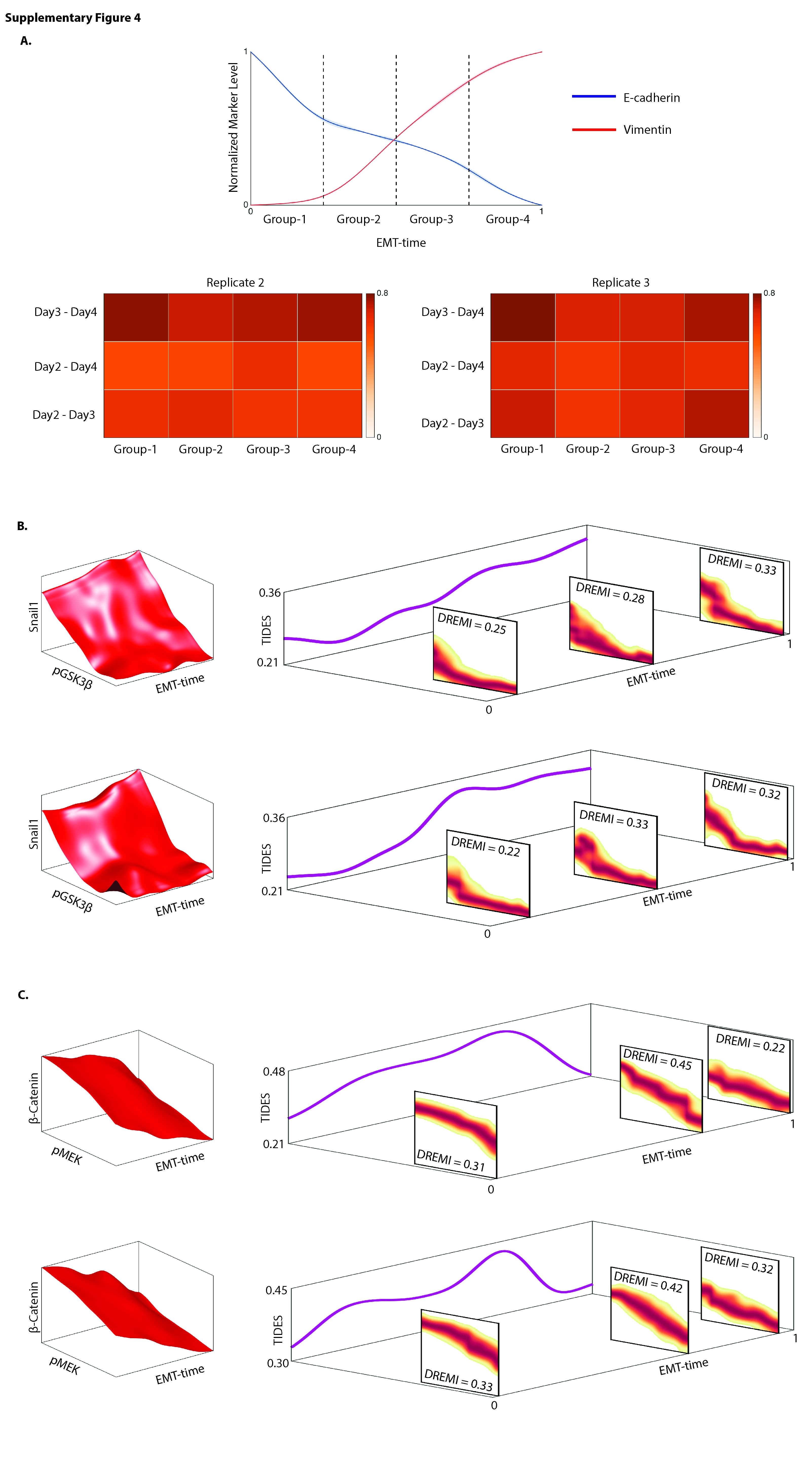

Supplement: S4 Fig — Signaling relationships along EMT-time in replicates: (A) TGFβ-treated cells from Days 2, 3 and 4 are binned into four groups along EMT-time. DREMI score between all pairs of signaling molecules is computed in each group. Heat map shows the correlation of the DREMI scores for each group across days. Average correlation is 0.68 (Replicate-2) and 0.73 (Replicate-3). (B) Dynamics of the relationship between pGSK3β and Snail1, similar to main Fig 3D across biological replicates. 3D-DREVI depicts the typical expression of Snail1 conditioned on pGSK3β and EMT-time. The modulation in the relationship is visualized by the 2D-DREVI slices along EMT-time and quantified the TIDES curve (purple curve) shown along the z-axis. (C) Dynamics of the relationship between pPLCγ2 and pMEK1/2 similar to Fig 3E across biological replicates. (TIFF) [file pone.0203389.s004.tiff]

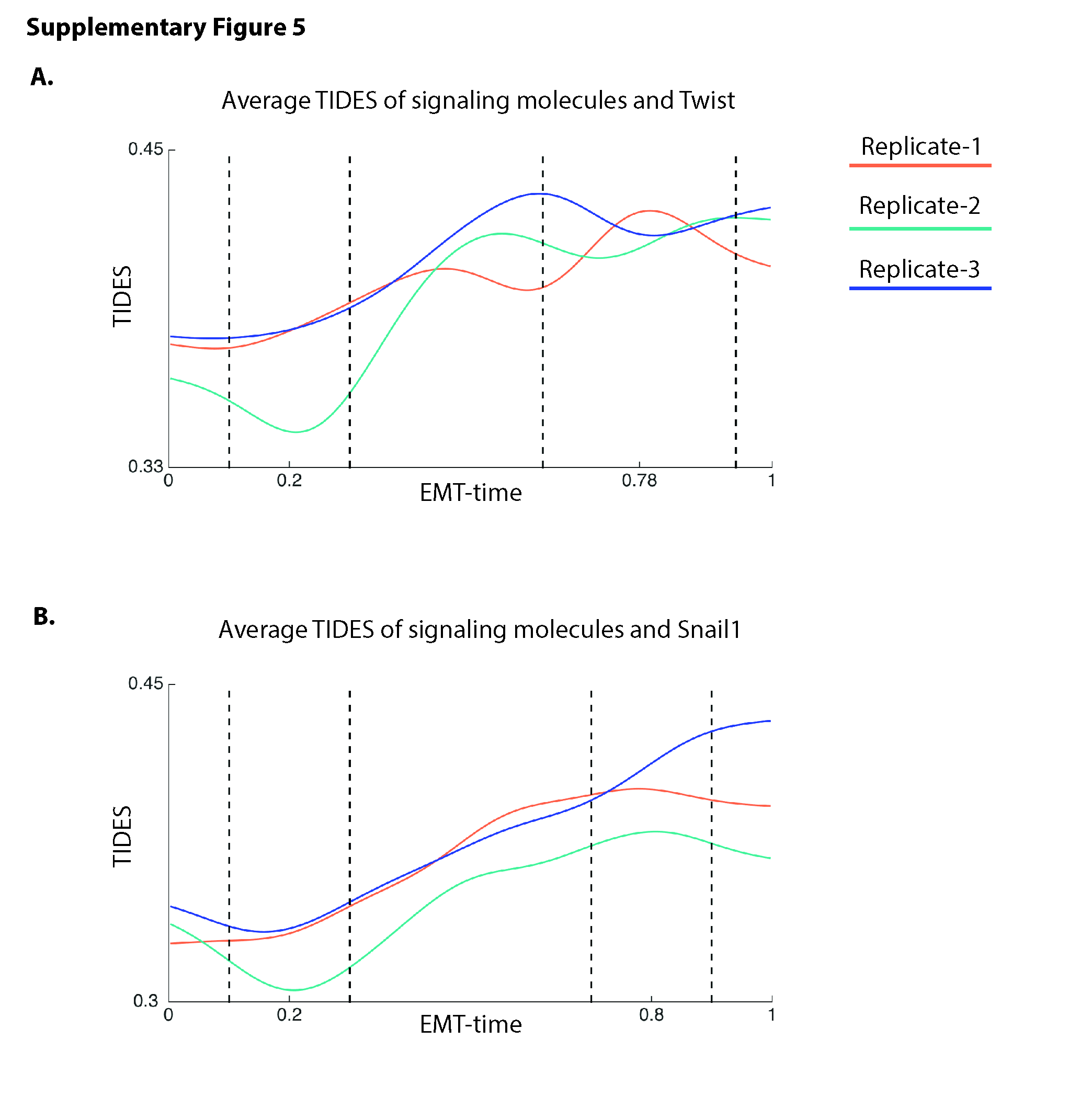

Supplement: S5 Fig — Information transfer during EMT across transcription factors: Average TIDES curve of the relationship between several molecules (pCREB, pSTAT5, pFAK, pMEK1/2, pNFκB, pP38, pAMPK, pAKT, pERK1/2, pGSK3β, pSMAD1/5, pSMAD2/3, β-catenin, CAH IV, pMARCK, pPLCγ2, pS6, pSTAT3) and Snail1 (B) and Twist (C), across three replicates for Day 3. Similar to Slug in main Fig 4, the curves start rising steadily at near EMT-time ~ 0.25, and peak near EMT-time ~ 0.75. (TIFF) [file pone.0203389.s005.tiff]

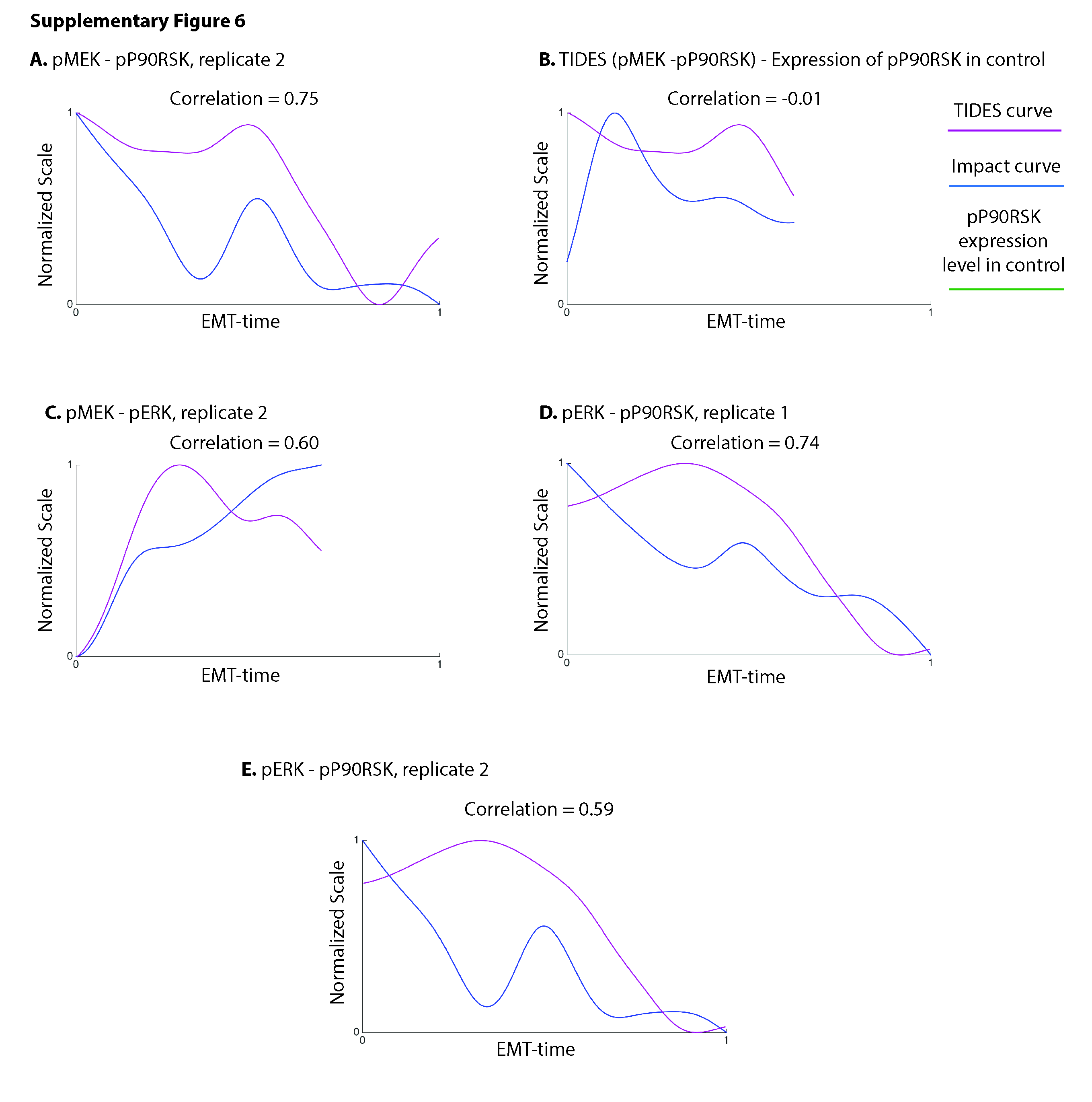

Supplement: S6 Fig — Validation of TIDES via short-term drug inhibition for direct and indirect edges in replicates: (A) Cross-correlation of TIDES curve between pMEK1/2-pP90RSK with the impact curve of pP90RSK results in a high correlation. This is a biological replicate of main Fig 5A. (B) Cross-correlation of TIDES curve between pMEK1/2-pP90RSK with the expression level of pP90RSK under control. Lower correlation than in (A) indicates that TIDES does not trivially follow the levels of pP90RSK. The curves end at EMT-time ~0.5 as the control does not contain sufficient cells in the mesenchymal state. (C) Biological replicate of Fig 5B; cross-correlating TIDES curve between pMEK1/2-pERK1/2 with the impact curve of pERK1/2 results in a high correlation. (D)-(E) Cross-correlation of pERK1/2-pP90RSK TIDES curve and pP90RSK impact curve under MEK-inhibition is 0.84 and 0.80 across two replicates. (TIFF) [file pone.0203389.s006.tiff]

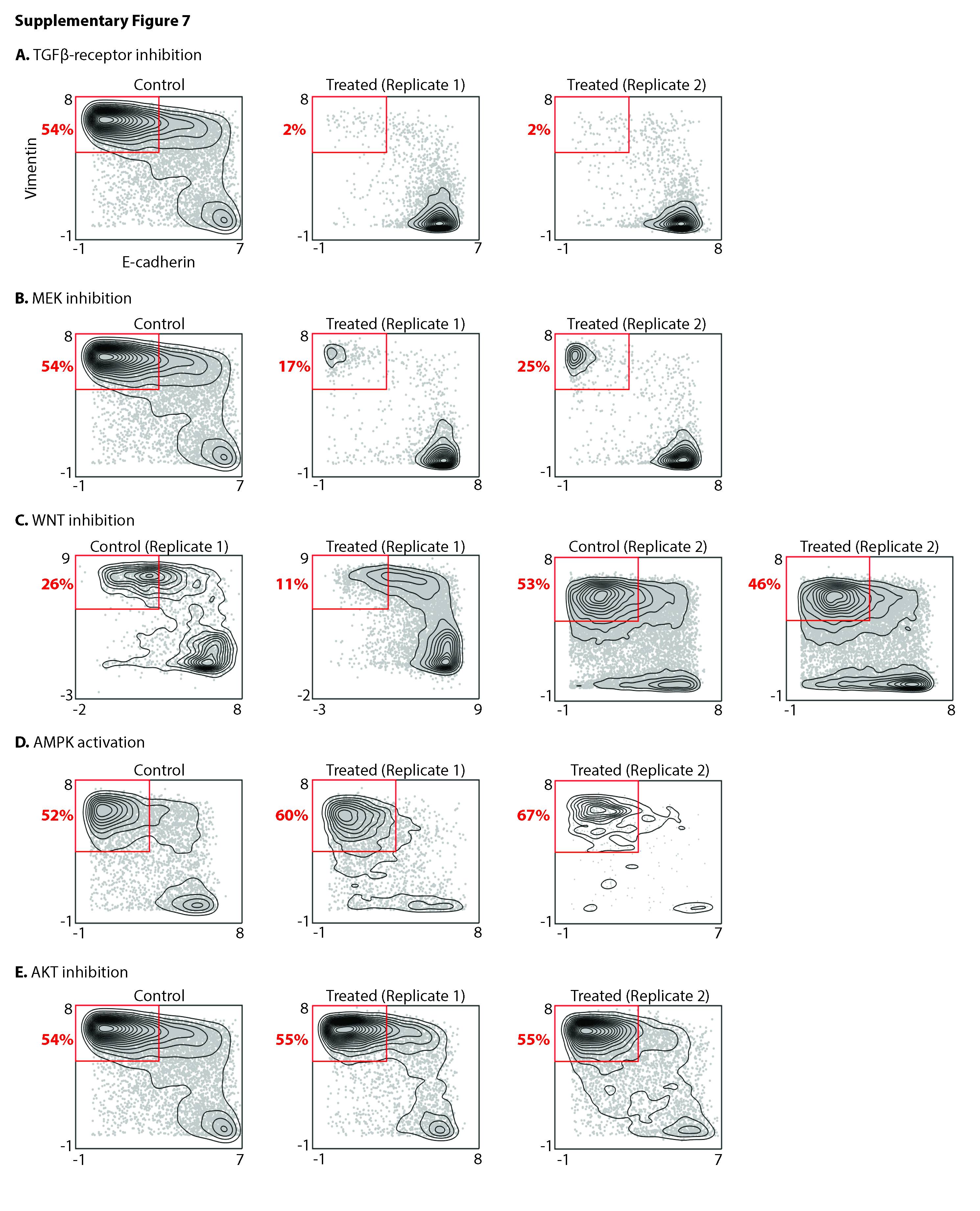

Supplement: S7 Fig — Validation of critical edges for EMT via long-term drug inhibition in replicates: (A)-(E) Shown are contour plots of cells treated with TGFβ (Control) and with TGFβ plus a chronic drug perturbation of the stated molecule for 5 Days, across biological replicates. Results of replicate 1 were shown as bar plots in Fig 6. Inhibition of TGFβ-receptor (A), MEK (B) and WNT (C) cause a substantial decrease in the fraction of cells that complete transition, while activation of AMPK (D) increases the proportion of cells that complete transition. AKT (E) on the other hand does not seem to impact the transition. (TIFF) [file pone.0203389.s007.tiff]

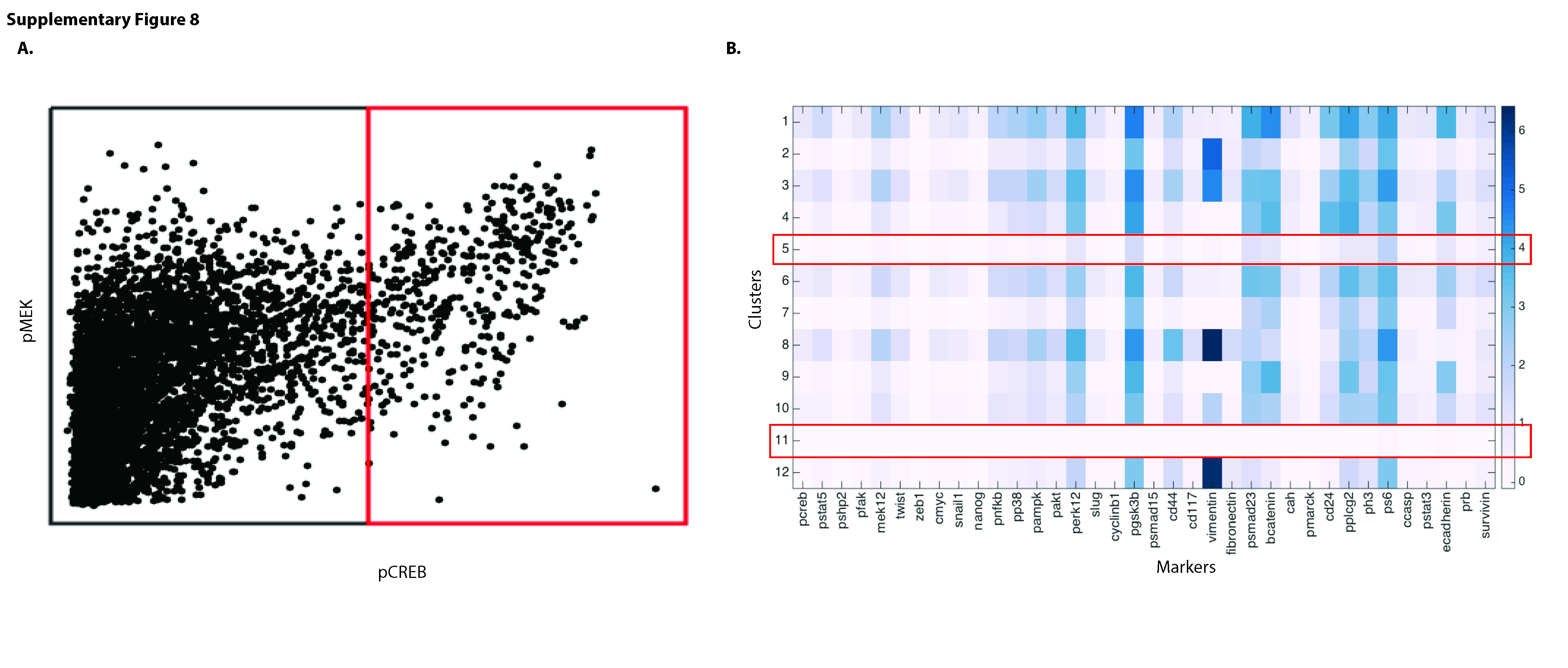

Supplement: S8 Fig — Data clean-up: (A). Scatterplot showing the relationship between pCREB and pMEK1/2 on Day 3 (shown is replicate 1). A spurious relationship between pCREB and pMEK1/2 at high pCREB values is seen. These events were manually gated out from time course and acute inhibition validation data sets. (B) Shown are heat maps of the expression of markers on various clusters obtained using Phenograph [46] on a set of phenotypic markers and transcription factors. The data shown is from Day 3 (replicate 1). The cells comprising the clusters with low expression of markers (such events are found in most mass cytometry experiments) were removed (indicated by red rectangles) from further analysis. (TIFF) [file pone.0203389.s008.tiff]

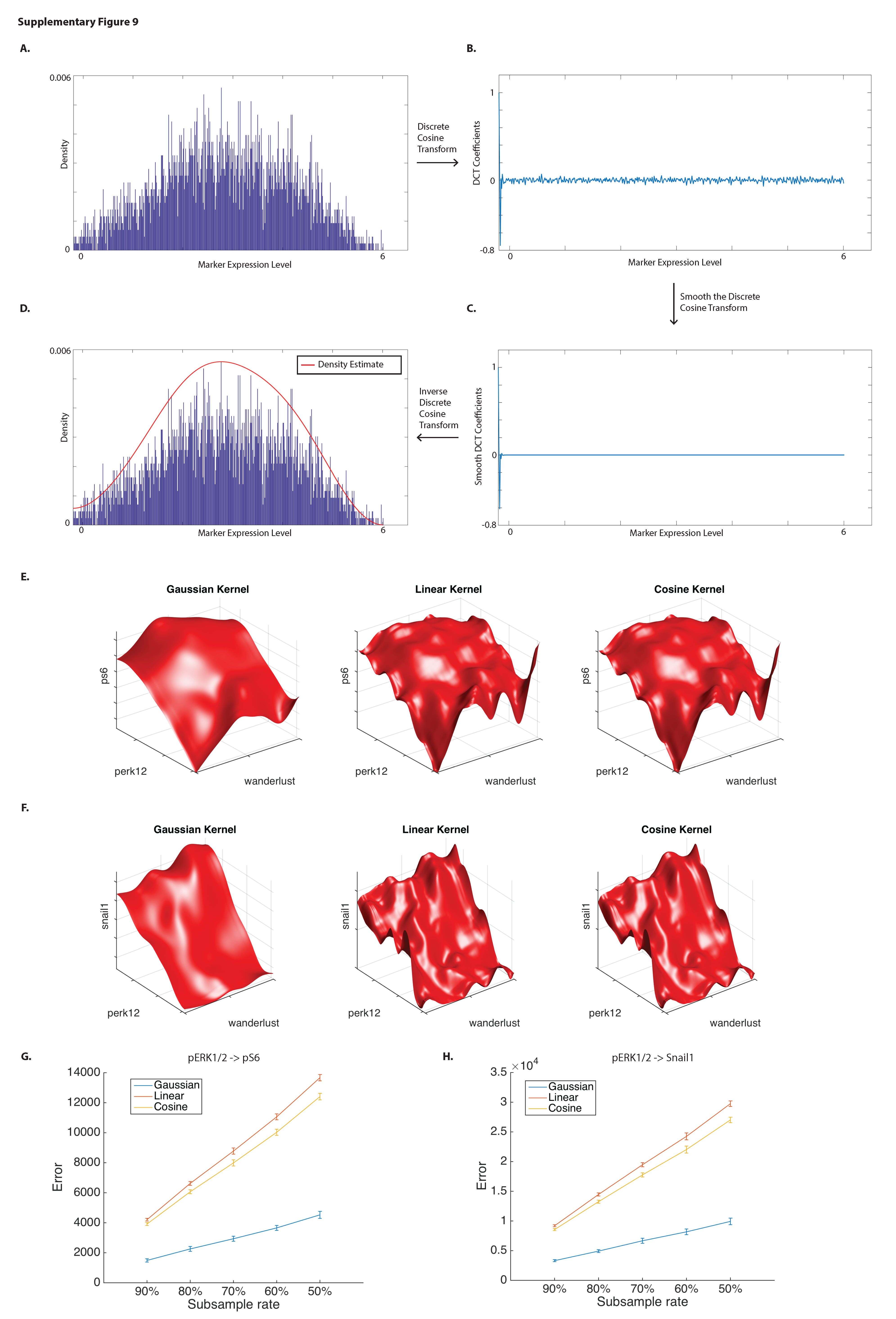

Supplement: S9 Fig — Computing Kernel Density Estimate: (Α) Plot shows histogram of a randomly chosen marker on Day 3. Constructing the histogram of the data is the first step in computing kernel density estimate. The histogram represents the initial condition for solving the heat equation. (Β) The histogram is then transformed into frequency domain by the Discrete Cosine Transform (DCT). (C) A low-pass filter smooths the DCT by removing the noisy parts. This is obtained by multiplying the DCT by an exponentially decaying term (exp(-k2π2t/2), see Methods) or in other words, evolving the initial condition in time. (D) The smooth density estimate is derived by inverting the smoothed-DCT. (E) Plot shows 3D-DREVI of pERK1/2 -> pS6 along EMT-time for various choices of kernel (Gaussian, linear and cosine) used in the estimation of underlying 3D-density. (F) Same as (E) for pERK1/2 -> Snail1 along EMT-time. (G) Plot shows robustness of each of the kernels against various sub samplings of the data. The density estimated using the full data is taken as the ground truth and the error is defined as the sum of the absolute difference between density estimated in various subsamples of the data against the ground truth. The solid line indicates the mean and the error bars indicate 1-standard deviation across 20 repetition of subsampling. The result is shown for pERK1/2 -> pS6 along EMT-time using Day 3 Replicate 1 data. (H) Same as (G) for pERK1/2 -> Snail1 along EMT-time (see Methods). (TIFF) [file pone.0203389.s009.tiff]

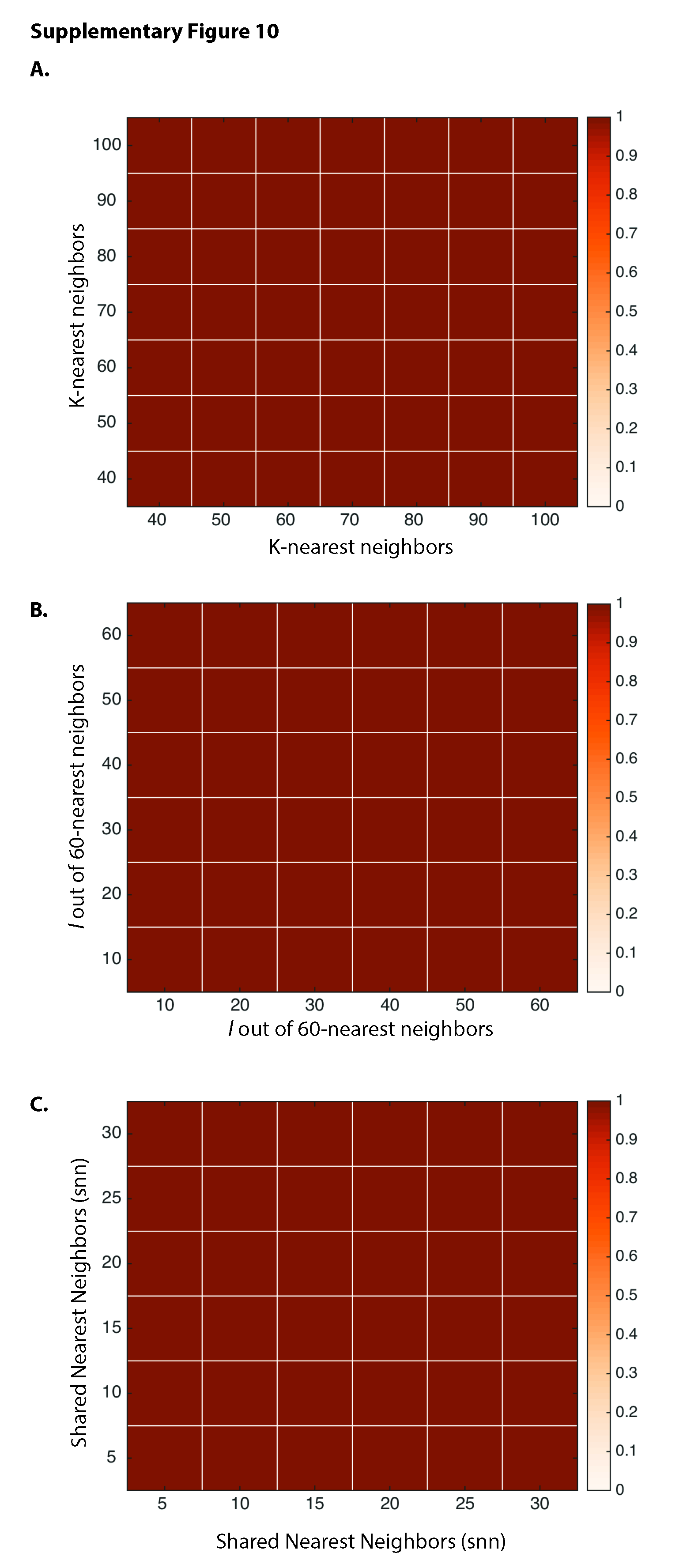

Supplement: S10 Fig — Robustness of Wanderlust generated EMT-time: (A) Heat-map shows the correlation between trajectories generated for various values of K-nearest neighbors. The shared nearest neighbor (snn) parameter was fixed at 20, while the l parameter (to avoid short circuits in the graph) was fixed at 12. (B) Heat-map shows the correlation between trajectories generated for various values of l parameter. K was fixed at 60 and snn was fixed at 20. (C) Heat-map shows the correlation between trajectories generated for various values of snn. K was fixed at 60 and l was fixed at 12. The results shown are for data on Day 3 (replicate 1), and holds true for all of our data. (TIFF) [file pone.0203389.s010.tiff]

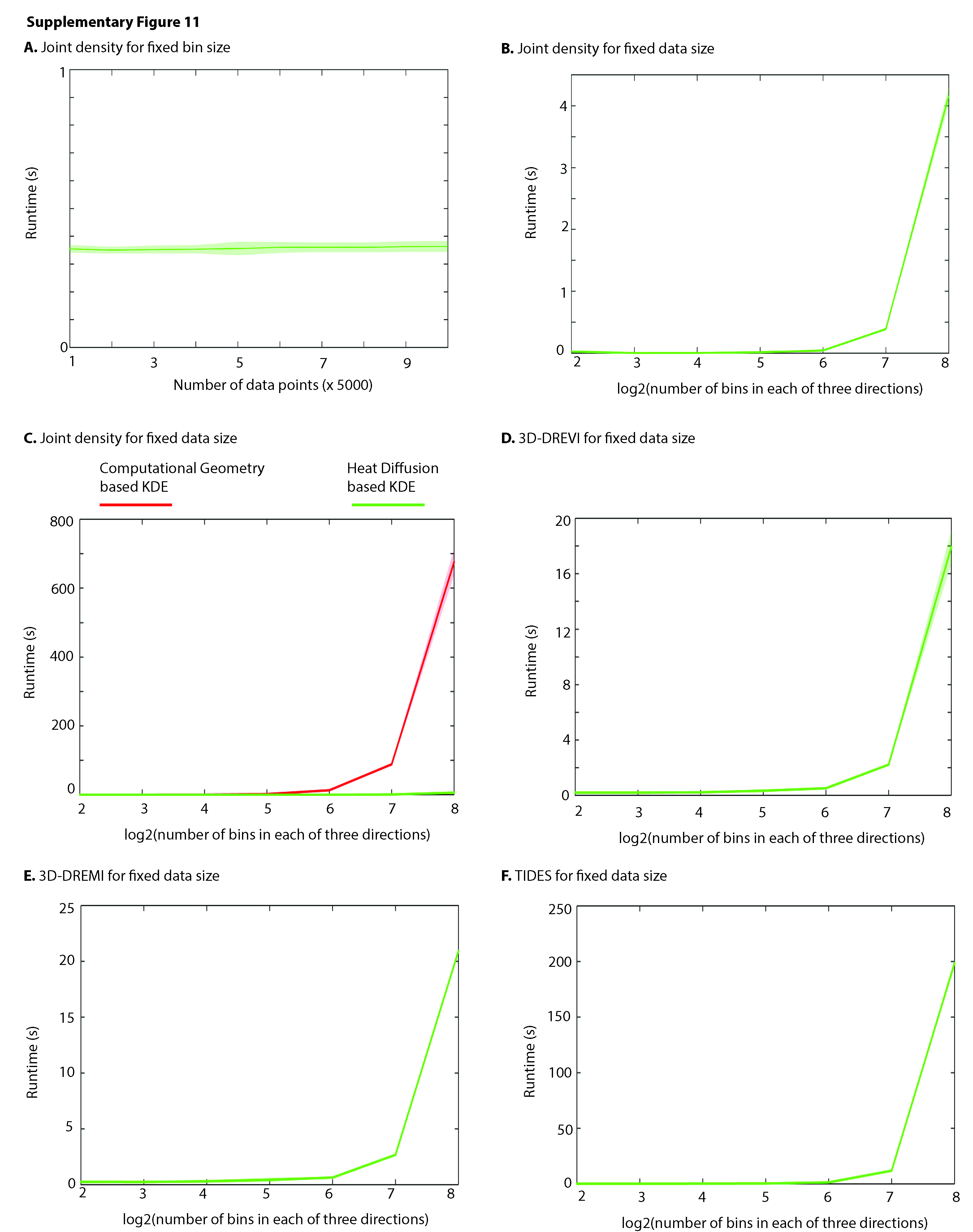

Supplement: S11 Fig — (A) Runtime for computing heat-diffusion based KDE against data size. The data is uniformly generated with 3 features. The number of bins used to construct the initial histogram was fixed to 128 in each of the three directions. The green line shows the mean while shaded region shows one standard deviation for 100 iterations. (B) Runtime against the log of number of histogram bins (in each of the three directions) for 5000 uniformly generated points. Computing density estimates on 224 points takes less than 10 seconds. (C) Runtime comparison of our method against an alternate (based on computational geometry) [47] for computing three-dimensional KDE. The shaded region shows the standard deviation across three replicates from the time-course data Day 3 for an edge (pS6-pGSK3β) and EMT-time. Our method can compute 3D density estimates at 224 points in less than 10 seconds while the alternative takes more than 10 minutes. (D)-(F) Plots show runtime of heat-diffusion based method against log of number of bins in computing 3D-DREVI, 3D-DREMI and TIDES respectively. The shaded region shows the standard deviation in runtime across three replicates of data from Day 3 for the edge used in (C), and the middle line shows the mean runtime. (TIFF) [file pone.0203389.s011.tiff]

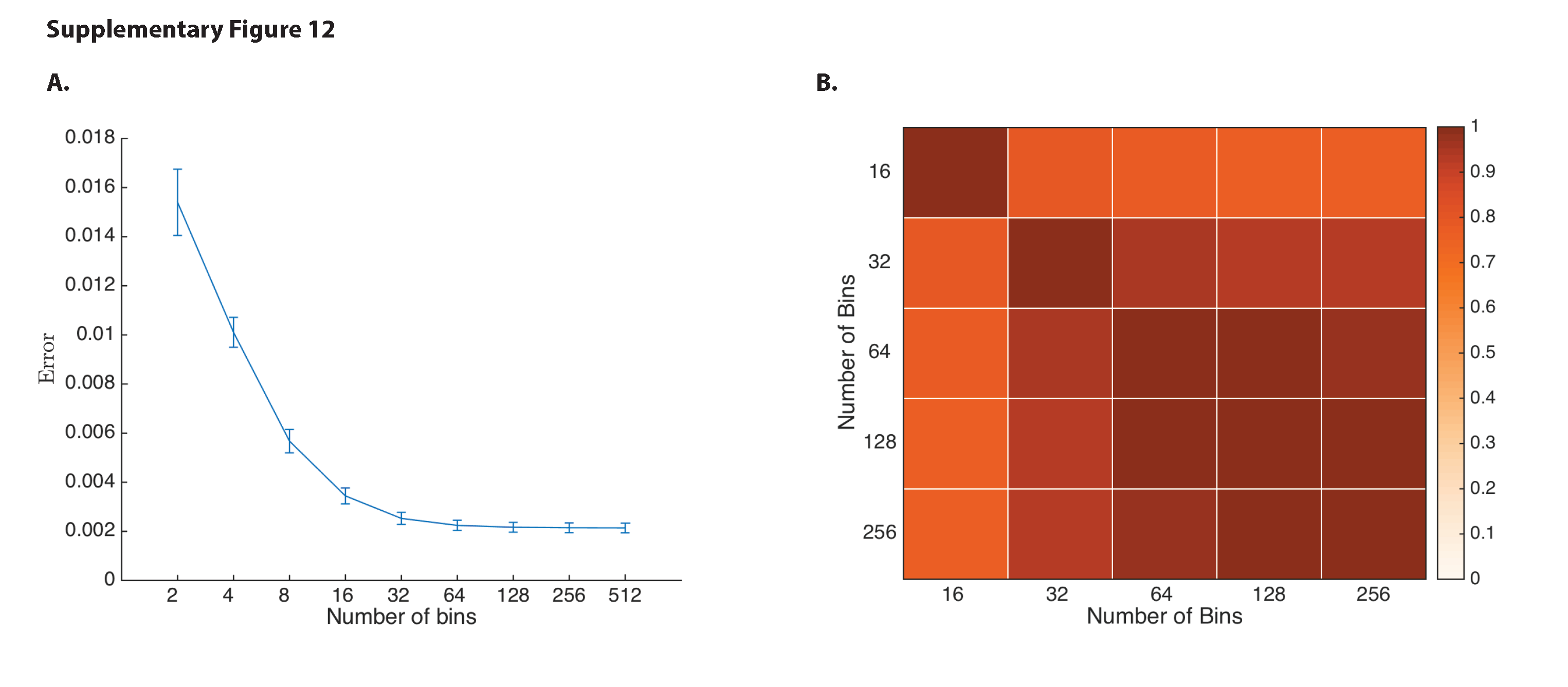

Supplement: S12 Fig — Performance Analysis: (A) Performance analysis of the presented method against number of bins. The plot shows reconstruction error against number of bins on a simulated data. The underlying distribution is a 3-dimensional Gaussian distribution with mean 0.5 and variance 0.3 in each of X, Y and Z directions. The reconstruction error is defined as the sum of absolute difference between the ground truth and the estimated density (using our method) for various number of bins normalized by the number of points density is estimated at (nbins x nbins x nbins). The density was estimated using 5000 randomly sampled points from the underlying distribution. The solid line shows the mean and the error bars show 1-standard deviation across 20 random sampling of the data points. (B) Plot shows the correlation in TIDES scores computed using various number of bins and averaged over a set of 100 randomly chosen pairs of proteins from Day3 Replicate 1 data. (TIFF) [file pone.0203389.s012.tiff]
