# Supplementary material for: Learning time-varying information flow from single-cell epithelial to mesenchymal transition data
Source: PLoS One. 2018 Oct 29;13(10):e0203389. doi: 10.1371/journal.pone.0203389 (PMC6205587; doi:10.1371/journal.pone.0203389)
Supplement: S1 Table — (DOCX) [file pone.0203389.s013.docx]

**Table S1:**

| **Isotope** | **Antibody** | **Clone** | **Concentration [µg/ml]** | **Supplier** |
| --- | --- | --- | --- | --- |
| La-139 | CREB (pSer133) | J151-21 | 2 | BD |
| Pr-141 | STAT5 (pTyr694) | 47/STAT5 | 5 | BD |
| Nd-142 | SHP2 (pTyr580) | D66F10 | 4 | CST |
| Nd-143 | FAK (pTyr397) | Polyclonal | 2.5 | CST |
| Nd-144 | MEK1/2 (pSer221) | 166F8 | 4 | CST |
| Nd-145 | Twist | poly ABD29 | 4 | Millipore |
| Nd-147 | c-MYC | D84C12 | 4 | CST |
| Nd-148 | Snail | ab180714 | 5 | Abcam |
| Nd-150 | NFκB p65 (pSer529) | polyclonal 9 | 3 | Abcam |
| Eu-151 | P38 (pThr180/pTyr182) | 36/p38 | 4 | BD |
| Sm-152 | AMPK (pThr172) | 40H9 | 4 | CST |
| Eu-153 | AKT (pSer473) | D9E | 5 | CST |
| Sm-154 | ERK1/2 (pThr202/pTyr204) | 20A | 2 | CST |
| Gd-155 | Slug | 666633 | 3 | R&D |
| Gd-156 | CyclinB1 | GNS-11 | 6 | BD |
| Gd-158 | GSK3ß (pSer9) | D85E12 | 1 | CST |
| Tb-159 | SMAD1/5 (pSer463/pSer465) | 41D10 | 6 | CST |
| Gd-160 | CD44 | IM7 | 0.01 | BD |
| Dy-161 | Vimentin | D21H3 | 1 | CST |
| Dy-163 | SMAD2/3 (pSMAD2(Ser465/67)/pSMAD3 (Ser423/425)) | D27F4 | 2 | CST |
| Dy-164 | β-Catenin | D13A1 | 2 | CST |
| Ho-165 | CAH IV | AF2188 | 4 | R&D |
| Er-167 | MARCK (pSer167/pSer170) | D13E4 | 4 | CST |
| Er-168 | CD24 | 30-F1 | 3 | Biolegend |
| Tm-169 | PLCγ2 (pTyr759) | K86-689.37 | 5 | BD |
| Er-170 | Histone H3 (pSer28) | HTA28 | 1 | Biolegend |
| Yb-171 | S6 (pSer235/pSer236) | N7-548 | 2 | BD |
| Yb-172 | Cleaved Caspase 3 | C92-605 | 5 | CST |
| Yb-173 | STAT3 (pTyr405) | 4/p-stat3 | 5 | BD |
| Yb-174 | E-cadherin | 36/E-cadherin | 1 | BD |
| Lu-175 | Rb (pSer807/pSer811) | D20B12 | 4 | CST |
| Yb-176 | Survivin | 71G4B7 | 4 | CST |
