# Supplementary material for: Learning time-varying information flow from single-cell epithelial to mesenchymal transition data
Source: PLoS One. 2018 Oct 29;13(10):e0203389. doi: 10.1371/journal.pone.0203389 (PMC6205587; doi:10.1371/journal.pone.0203389)
Supplement: S2 Table — (DOCX) [file pone.0203389.s014.docx]

**Table S2:**

| E-cadherin |
| --- |
| Vimentin |
| CD44 |
| β-catenin |
| Snail1 |
| Slug |
