# Supplementary material for: Learning time-varying information flow from single-cell epithelial to mesenchymal transition data
Source: PLoS One. 2018 Oct 29;13(10):e0203389. doi: 10.1371/journal.pone.0203389 (PMC6205587; doi:10.1371/journal.pone.0203389)
Supplement: S3 Table — (DOCX) [file pone.0203389.s015.docx]

**Table S3:**

| **X-molecule** | **Y-molecule** | **Average score** |
| --- | --- | --- |
| pSMAD2/3 | β-catenin | 0.967 |
| pAMPK | β-catenin | 0.891 |
| ERK1/2 | β-catenin | 0.886 |
| pGSK3β | β-catenin | 0.874 |
| pGSK3β | pERK1/2 | 0.866 |
| pMEK1/2 | β-catenin | 0.863 |
