# Supplementary material for: Learning time-varying information flow from single-cell epithelial to mesenchymal transition data
Source: PLoS One. 2018 Oct 29;13(10):e0203389. doi: 10.1371/journal.pone.0203389 (PMC6205587; doi:10.1371/journal.pone.0203389)
Supplement: S4 Table — (DOCX) [file pone.0203389.s016.docx]

**Table S4:**

| **Target** | **Small molecule** |
| --- | --- |
| TGFβ-R1 | SB431542 |
| MEK | PD318088 |
| WNT | XAV-939 |
| AMPK | Phenformin |
| AKT | PHT427 |
